# Dominant integration locus drives continuous diversification of plant immune receptors with exogenous domain fusions

**DOI:** 10.1186/s13059-018-1392-6

**Published:** 2018-02-19

**Authors:** Paul C. Bailey, Christian Schudoma, William Jackson, Erin Baggs, Gulay Dagdas, Wilfried Haerty, Matthew Moscou, Ksenia V. Krasileva

**Affiliations:** 1 Earlham Institute, Norwich Research Park, Norwich, NR4 7UZ UK; 2 The Sainsbury Laboratory, Norwich Research Park, Norwich, NR4 7UH UK

**Keywords:** Plant immunity, Disease resistance genes, NLRs, Gene fusions

## Abstract

**Background:**

The plant immune system is innate and encoded in the germline. Using it efficiently, plants are capable of recognizing a diverse range of rapidly evolving pathogens. A recently described phenomenon shows that plant immune receptors are able to recognize pathogen effectors through the acquisition of exogenous protein domains from other plant genes.

**Results:**

We show that plant immune receptors with integrated domains are distributed unevenly across their phylogeny in grasses. Using phylogenetic analysis, we uncover a major integration clade, whose members underwent repeated independent integration events producing diverse fusions. This clade is ancestral in grasses with members often found on syntenic chromosomes. Analyses of these fusion events reveals that homologous receptors can be fused to diverse domains. Furthermore, we discover a 43 amino acid long motif associated with this dominant integration clade which is located immediately upstream of the fusion site. Sequence analysis reveals that DNA transposition and/or ectopic recombination are the most likely mechanisms of formation for nucleotide binding leucine rich repeat proteins with integrated domains.

**Conclusions:**

The identification of this subclass of plant immune receptors that is naturally adapted to new domain integration will inform biotechnological approaches for generating synthetic receptors with novel pathogen “baits.”

**Electronic supplementary material:**

The online version of this article (10.1186/s13059-018-1392-6) contains supplementary material, which is available to authorized users.

## Background

Plants have powerful defense mechanisms that rely on an arsenal of plant immune receptors [1, 2]. Nucleotide binding leucine rich repeat (NLR) proteins represent one of the major classes of plant immune receptors. Plant NLRs are modular proteins characterized by a common NB-ARC domain similar to the NACHT domain in mammalian immune receptor proteins [1]. On the population level, NLRs provide plants with sufficient diversity to maintain immunity to rapidly evolving pathogens [3, 4]. Recent findings show that novel pathogen recognition specificities can also be acquired through the fusion of non-canonical domains to NLRs [5–7] and that such fusions are widespread across flowering plants [8, 9]. These exogenous domains can serve as “baits” mimicking host targets of pathogen-derived effector molecules [5, 6, 10].

Well-studied cases of NLRs with integrated domains (NLR-IDs) include *Arabidopsis thaliana RRS1* (*NLR-WRKY*) and *Oryza sativa RGA5* (*NLR-HMA*). Both NLR-IDs require an additional genetically linked NLR, *RPS4*, and *RGA4*, respectively, for the activation of disease resistance [5, 10, 11]. The *RGA4/RGA5* and *RRS1/RPS4* pairs are found as neighboring genes in inverse orientation and share a common promoter suggesting co-regulation. Paralogs of *RRS1* in *Arabidopsis* also require an additional NLR partner [11, 12]. The products of paired NLRs form protein complexes that suppress NLR auto-activation. While the NLR-ID is responsible for initial effector perception, its NLR partner is required for downstream signaling [5, 6, 10]. Whether NLR-IDs always require a genetically linked partner remains unclear.

NLR-IDs represent a successful use of genetic and protein linkage of NLRs with other genes to expand and diversify the pathogen recognition repertoire. On average, 10% of NLRs in sequenced plant species have been shown to contain exogenous integrated domains [8, 9]. However, little is known about the mechanisms and evolutionary history underlying NLR-ID formation.

The availability of sequenced genomes facilitates analyses of the evolution and diversification of NLR-IDs. The *Poaceae* (grasses) are a highly successful family of flowering plants that originated 120 million years ago [13, 14]. This family includes the three major cereals in modern day agriculture and human diet: maize (*Zea mays*), rice (*O. sativa*), and wheat (*Triticum* spp.). It has been suggested that the high genomic plasticity of grasses contributed to their adaptability and success in agriculture [15]. The genomes of sequenced grasses range in size from 270 Mb for *Brachypodium distachyon* to 17 Gb for the hexaploid bread wheat (*T. aestivum*) and differ in chromosome number and ploidy level [16]. The genomes of grasses acquired diverse variation in gene copy number, including a high copy number of NLRs [9, 17, 18], making the *Poaceae* family an attractive system to study NLR evolution.

We examined the evolutionary dynamics of NLR-IDs in the genomes of nine grass species to address the following questions. First, were NLR-IDs distributed uniformly across subclasses of NLRs or were there specialized clades more prone to exogenous domain integration? Second, we asked what was the molecular mechanism underlying NLR-ID formation. Previous sequence analysis of known NLR genes, such as *RGA5*, hinted at the diversity of integrated domains fused to their homologs; however, no evolutionary links between these genes have been established [7, 19].

We investigated the distribution of NLR-IDs within the NLR phylogeny and the diversity of their integrated domains within and across species. We identified several clades enriched in NLR-IDs including a monophyletic clade of NLRs that is highly amenable to repeated domain integrations from diverse gene families. The proteins within this clade showed significant lack of orthology and synteny conservation, providing evidence that orthologs acquired fusions to genes from diverse genomic locations. In addition, we identified a novel motif located upstream of integrated domains that is specifically associated with this clade and is maintained across diverse NLRs. Uncovering the diversity of IDs can form the basis for new biotechnological approaches towards designing NLR receptors with synthetic fusions to new pathogen traps.

## Results and Discussion

### NLR-IDs are distributed unevenly across the NLR phylogeny with one dominant clade containing a diverse set of new integrations

We examined the evolution of NLRs and NLR-IDs across nine grass species with available genomes: *Setaria italica*; *Sorghum bicolor*; *Z. mays*; *B. distachyon*; *O. sativa*; *Hordeum vulgare* (barley); *Aegilops tauschii*; *Triticum urartu*, and *Triticum aestivum* (hexaploid bread wheat). These genomes are assembled and annotated at similar quality as indicated by Benchmarking Universal Single-Copy Orthologs (BUSCO) analyses (Additional file 1).

We tested two non-exclusive hypotheses about NLR-IDs:

1. The integration of exogenous domains occurs at random during NLR evolution.
2. There are conserved evolutionary integrations facilitating NLR-ID diversification.

**Fig. 1:**
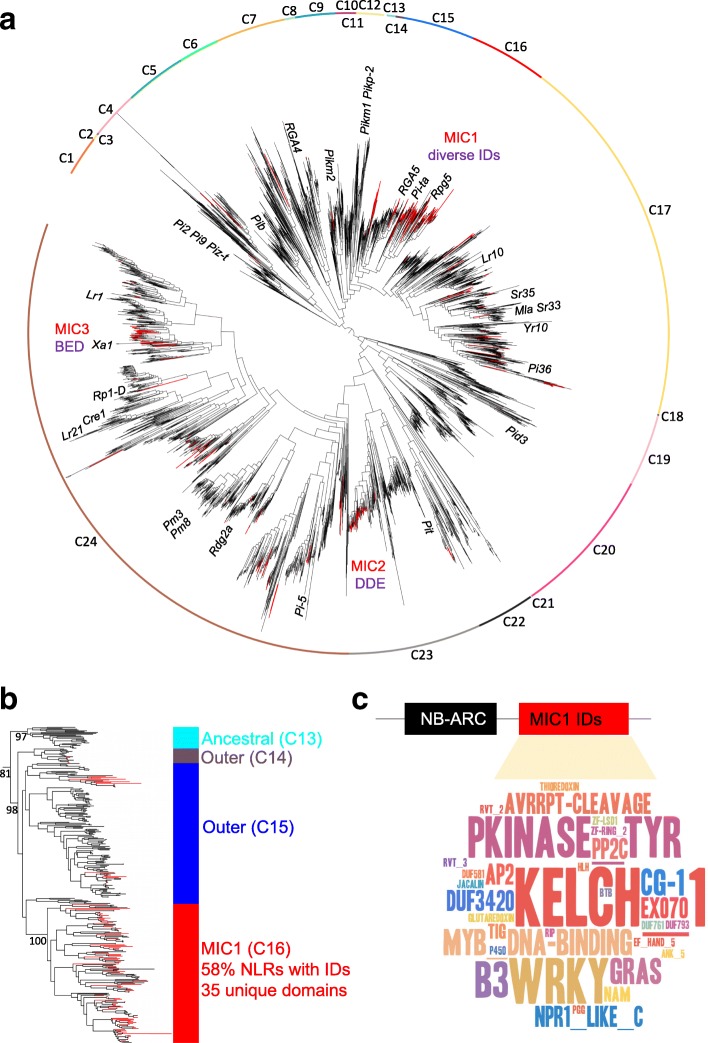
Maximum likelihood phylogeny of NLRs in grasses identifies evolutionary hotspots of NLRs with integrated domains. **a** The maximum likelihood tree of the NB-ARC family in grasses (4184 proteins, nine species) showing occurrence of integrated domain (ID) across the phylogeny (*red branches*). **b** Close-up of MIC1 (*red*) as well as its outgroup clades C15 (*blue*), C14 (*brown*), and ancestral clade C13 (*cyan*) showing the key bootstrap support values. **c** Wordcloud summary of the integrated domain diversity from MIC1. E-value cut-off for presence of an ID domain, 0.001

We constructed a maximum likelihood phylogenetic tree of 4184 NLRs from these species, based on the common NB-ARC domain. The resulting phylogeny was sub-divided into 24 distinct clades C1 to C24 based on high bootstrap support and branch length (BRL) information (Fig. 1a, Additional file 2). We observed that while NLR-IDs occurred at low frequency across the full phylogeny, a small subset of clades had a much higher proportion of NLR-IDs (Fig. 1a, Table 1, Additional file 2). We called these clades major integration clades (MICs) 1, 2, and 3 (Fig. 1a). MIC1 (C16) accounted for nearly 30% of all NLR-IDs present in the phylogeny (Table 1). Across nine species, on average, 58% of NLRs in MIC1 have integrated domains compared to 8% across all clades (Fig. 1b, Table 1). We might expect this number to be even higher with improved assemblies and annotations. MIC1 was nested within an outer clade (Fig. 1b, C15, highlighted in blue) with only 13% NLR-IDs.

**Table 1:** Number of NLRs and NLR-IDs in MIC1, MIC2, and MIC3 in nine grass species

	Fig. 1a tree				Root clade (C13)			Outer clade (C14, C15)			Inner MIC1 (C16)				Outer clade to MIC2 (C23)			Inner MIC2 (C23)			MIC3 (C24)		
Species	Total	+ID^a^	% + ID	Total	Total	+ID	% + ID	Total	+ID	% + ID	Total	+ID	% + ID	Total	Total	+ID	% + ID	Total	+ID	% + ID	Total	+ID	% + ID
*S. italica*	281	9	2.96	13	1	0	0	5	0	0.00	7	2	28.57	11	10	0	0.00	1	0	0.00	4	2	50.00
*S. bicolor*	262	22	8.40	19	3	0	0	10	0	0.00	6	4	66.67	11	2	0	0.00	9	0	0.00	3	3	100.00
*Z.mays*	84	7	8.33	1	1	0	0	0	0	0.00	0	0	0.00	2	1	0	0.00	1	0	0.00	0	0	0.00
*O. sativa*	362	18	4.97	17	3	0	0	7	0	0.00	7	3	42.86	8	8	0	0.00	0	0	0.00	4	2	50.00
*B. distachyon*	255	16	6.27	25	3	0	0	10	0	0.00	12	7	58.33	8	1	0	0.00	7	4	57.14	4	3	75.00
*H. vulgare*	311	27	8.68	36	2	0	0	12	0	0.00	22	13	59.10	8	4	1	25.00	4	1	25.00	6	4	66.67
*A. tauschii* (D)	482	67	13.90	47	3	0	0	28	4	14.29	16	12	75.00	20	4	1	25.00	16	0	0.00	11	9	81.82
*T. aestivum* (A, B and D)	1732	133	7.68	166	7	0	0	91	13	14.29	68	40	58.82	62	18	1	5.56	44	27	61.36	35	12	34.29
*T. aestivum* (A only)	513	35	6.82	61	0	0	0	33	3	9.09	28	16	57.14	17	6	0	0.00	11	6	54.55	10	2	20.00
*T. aestivum* (B only)	619	50	8.08	56	4	0	0	33	7	21.21	19	11	57.89	24	6	0	0.00	18	10	55.56	8	5	62.50
*T. aestivum* (D only)	497	34	6.84	45	3	0	0	23	2	8.70	19	11	57.89	12	6	1	16.67	6	3	50.00	13	4	30.77
ChrU only	103	14	13.59	4	0	0	0	2	1	50.00	2	2	100.00	9	0	0	0.00	9	8	88.89	4	1	25.00
*T. urartu* (A)	377	32	8.49	38	2	0	0	17	6	35.29	19	10	52.63	4	1	0	0.00	3	0	0.00	11	2	18.18
Total^b^	4146	331		362	25	0		180	23		157	91		134	49	3		85	32		78	37	
Average			7.94				0.00			12.78			57.96				6.12			37.65			47.44

^a^ + ID, proteins with integrated “ID” domain(s)

^b^Excludes sequences in Fig. 1a tree that are not from genome annotation

We examined the diversity of IDs in each clade to assess whether the high number of NLR-IDs in the major integration clades resulted from expansion of an ancestral integration (with the expectation of low ID diversity) or represents repeated integrations of different domains (high ID diversity). MIC2 and MIC3 showed low ID diversity and represented expansions of ancestral integrations of the DDE superfamily endonuclease and the BED-type zinc finger domains, respectively (Fig. 1a, Additional file 2). In contrast, the ID diversity in MIC1 was high (Fig. 1c) with its members harboring a total of 35 Pfam domains (Fig. 1c, Table 2).

**Table 2:** Summary of unique Pfam domains found in NLR-ID MIC1 (C16) clade and neighboring clades, C14 and C15

	Across all clades in tree		Neighboring clade (C14, C15)	MIC1 clade (C16)	
Species	Total NLR-ID genes (n)	Non-redundant ID domains (n)	Non-redundant ID domains (n)	Non-redundant ID domains (n)	Unique domains
*S. italica*	9	7	0	2	NAM, WRKY
*S. bicolor*	22	13	0	4	WRKY, HLH, NAM, Glutaredoxin
*Z. mays*	7	8	0	0	-
*O. sativa*	18	16	0	3	AvrRpt-cleavage, Thioredoxin, DUF761,
*B. distachyon*	16	9	0	7	AP2, Jacalin Myb_DNA-binding, Pkinase, Pkinase_Tyr, WRKY
*H. vulgare*	27	19	0	12	AvrRpt-cleavage, B3, DUF581, Exo70, Glutaredoxin, Kelch_1, PP2C, Pkinase, Pkinase_Tyr, PP2C WRKY, zf-LSD1
*A. tauschii* (D)	67	32	2	8	AvrRpt-cleavage, B3, Kelch_1, Pkinase, Pkinase_Tyr, RVT_2, WRKY, p450
*T. aestivum* subgenomes:	133	46	4	21	AP2, Ank_2, Ank_5, B3, BTB, CG-1, DUF3420, DUF793, Exo70, GRAS, Kelch_1, Myb_DNA-binding, NPR1_like_C, PGG, PP2C, Pkinase, Pkinase_Tyr, RIP, TIG, WRKY, zf-RING_2
A	35	20	2	13	
B	50	28	3	8	
D	34	23	2	13	
Unanchored	14	9	2	4	
*T. urartu* (A)	32	25	4	9	B3, CG-1, EF_hand_5, Exo70. Kelch_1, PP2C, Pkinase, Pkinase_Tyr, RVT_3
Average	37	19	1	7	-

Only domains with e-value < 1e-3 are shown. For the full list of domains with lower stringency (e-value < 0.05), see Additional file 2 and 14

We surveyed 38 well-studied monocot NLRs present in the phylogeny to see which of those were contained in MIC1 (Fig. 1a). Known resistance genes within MIC1 included *RGA5*, *Rpg5*, and *Pi-ta*, which encode NLR-HMA, NLR-kinase, and NLR-thioredoxin, respectively [10, 20–23].

### Proliferation of MIC1 NLRs in grasses is accompanied by continued domain shuffling

**Fig. 2:**
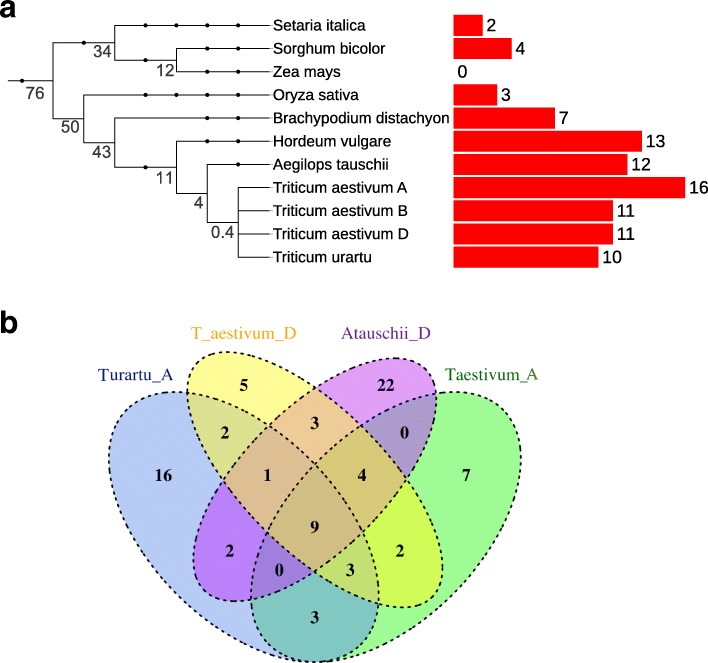
MIC1 has proliferated in grasses and continues to accumulate new domains as seen from comparison of wheat and its diploid progenitors. **a** Overall evolutionary relationship of grasses used in this study and corresponding number of NLR-IDs in MIC1. Key clade divergence is marked on the tree in millions of years as estimated at timetree.org. **b** The repertoires of IDs are different among wheat subgenomes and their progenitors suggesting continuous integration of new IDs

We examined the composition of NLR-IDs in MIC1 for each of the nine grass genomes in our study (Additional file 3). As diverse NLR-IDs were present in all the studied grass species with the exception of *Z. mays*, we postulate that this clade originated before the split of the BOP and PACMAD clades at least 76 million years ago [24]. Following the evolution of the *Pooideae* genomes, MIC1 seems to have expanded from two to four NLR-ID members in the genomes of rice, *Setaria* spp. and Sorghum to 7 to 16 NLR-ID in *Brachypodium* spp. and *Triticeae* spp. (Fig. 2a).

The allohexaploid nature of the wheat genome (A, B, D genomes) and availability of genomes from two diploid progenitors (A, *T. urartu* and D, *A. tauschii*) allowed us to further investigate if new integrations continued to occur in this lineage. We found that while the total numbers of NLR-IDs in the A and D genomes of *T. aestivum* relative to *T. urartu* and *A. tauschii* were highly similar (Fig. 2a), the ID diversity from the A, B, and D genomes of wheat and the A and D diploid progenitors were mostly non-redundant, indicating a continuous integration of new domains after the divergence of these species (Fig. 2b). It is possible that differences in the observed repertoires can be explained partly by incomplete genome annotations or fragmented assembly of NLRs in the diploid progenitors. However, the A, B, and D subgenomes of wheat are of the same quality and contain full-length NLRs [25], yet the ID repertoires among them are not fully overlapping. This suggests that the observed differences cannot be explained by varying assembly quality and new integrations are continuously occurring in this lineage. Moreover, the genome assemblies of *B. distachyon*, *Z. mays*, and *O. sativa* are of much higher quality than those of the *Triticeae* species (Additional file 1), yet they contain fewer NLR-IDs and have lower ID diversity (Table 1). In our later analyses (see “Results” section, “Duplication of genes encoding IDs followed by translocation of either ID or NLR lead to new NLR-ID formation”), we were able to identify homoeologous genes within wheat that contained distinct IDs. Altogether, these results suggest that integration of new domains in MIC1 NLRs is ongoing and results in diverse ID repertoires across species.

### NLR-IDs in MIC1 form genetic pairs with NLRs from another clade

**Fig. 3:**
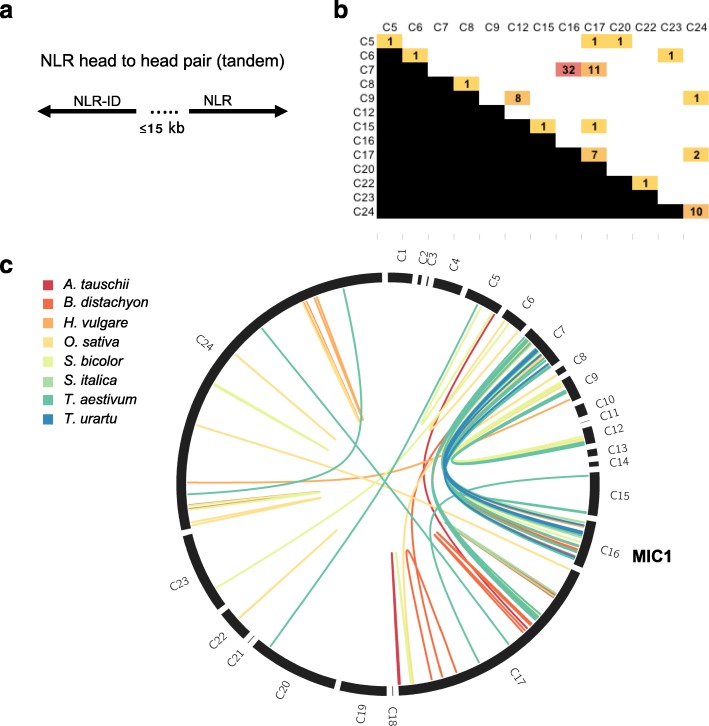
NLRs from MIC1 are genetically linked in head-to-head pairs with NLRs from clade C7. **a** The *diagram* shows the orientation and maximum distance of 15 kb that we used to identify NLR gene pairs. **b** *Heatmap* showing numbers of tandem NLRs across different clades. **c** *Circos plot* on showing links between NLRs from MIC1 (C16) and NLRs from other clades that are oriented across the plot in the same order as in the overall NLR phylogeny. NLR gene pair tandems are color-coded according to their species as indicated in the legend on the *left*

The NLR-IDs *RRS1* and *RGA5* require a genetically linked partner NLR *RPS4* and *RGA4*, respectively, to be functional. Homologs of *RRS1/RPS4* pair are also found in pairs [12]. We determined how many NLR-IDs from MIC1 and overall in the NLR phylogeny were paired with another NLR in head to head orientation (upstream NLR on the reverse strand, downstream NLR on the forward strand). Using our tandem analysis tool, we scanned the genome annotation for each species for such tandem-NLRs within a maximum distance of 15 kbp (Fig. 3a).

Our results showed that across all species (with the exception of *Z. mays*), tandem-NLRs are significantly enriched in the complete set of NLR-IDs (Table 3, Fig. 3b, Additional file 4). We found that 50 out of all 415 NLR-IDs (12.04%) are part of a tandem-NLR, in comparison to 268 out of 5779 (4.63%) of the NLRs without ID (*p* = 1.02e-08). Members of the MIC1 clade are also enriched in tandem NLRs, whether or not they contain an ID (*p* = 4.12e-12). We expect that the real number of tandems might be higher because most of the assemblies are still fragmented, especially in *A. tauschii* in which neighboring genes might not be detected due to short scaffolds [26].

**Table 3:** Gene pair (“tandem”) analysis reveals enriched occurrences of NLR-ID and MIC-1 tandems

	Tandems				NLRs			NLR-IDs		Other NLRs		
	Total	+NLR-ID	-NLR-ID	MIC1/NLR-ID	Total	NLR-IDs	Other	Tandem	Single	Tandem	Single	*p* value (Fisher’s exact)
*A. tauschii*	13	8 (1^a^)	4	3	738	83	655	10	73	16	639	2.24E-04
*B. distachyon*	22	5	17	4	372	19	353	5	14	39	314	6.01E-02
*H. vulgare*	10	3	7	2	462	27	435	3	24	17	418	1.04E-01
*O. sativa*	25	4	21	2	518	19	499	4	15	46	453	1.00E-01
*S. bicolor*	15	3 (1^a^)	11	1	341	24	317	5	19	25	292	4.79E-02
*S. italica*	11	1	10	1	438	12	426	1	11	21	405	4.66E-01
*T. aestivum*	48	17	31	10	2596	176	2420	17	159	79	2341	1.70E-04
*T. urartu*	13	5	8	3	558	43	515	5	38	21	494	4.17E-02
*Z. mays*	2	0	2	0	171	12	159	0	12	4	155	1.00E + 00
All	159	48	111	26	6194	415	5779	50	365	268	5511	1.02E-08
	Tandems				NLRs			MIC1-NLRs		Other NLRs		
	Total	+MIC1	-MIC1	MIC1/NLR-ID	Total	MIC1-NLRs	Other	Tandem	Single	Tandem	Single	*p* value (Fisher’s exact)
*A. tauschii*	13	4	9	3	738	47	691	4	43	22	669	7.66E-02
*B. distachyon*	22	8	14	4	372	25	347	8	17	36	311	4.69E-03
*H. vulgare*	10	4	6	2	462	36	426	4	32	16	410	6.11E-02
*O. sativa*	25	5	20	2	518	17	501	5	12	45	456	1.74E-02
*S. bicolor*	15	2	13	1	341	19	322	2	17	28	294	6.78E-01
*S. italica*	11	5	6	1	438	13	425	5	8	17	408	1.98E-04
*T. aestivum*	48	16 (1^b^)	31	10 (1^c^)	2596	166	2430	17	149	79	2351	8.13E-05
*T. urartu*	13	6	7	3	558	38	520	6	32	20	500	5.60E-03
*Z. mays*	2	0	2	0	171	1	170	0	1	4	166	1.00E + 00
All	159	51	108	26	6194	362	5832	52	310	266	5566	4.12E-12

Contingency table of discovered NLR tandems (head-to-head gene pairs within 15-kbp distance). Top: tandems involving NLR-IDs; bottom: tandems involving members of MIC1

MIC1- NLR-ID/NLR tandems generally consist of a MIC1-NLR-ID and a non-MIC1 NLR

^a^*A. tauschii* and *S. bicolor* have the only NLR-ID/NLR-ID tandems

^b^*T.aestivum* has one intra-MIC1 (outer clade) NLR/NLR tandem

^c^*T. aestivum* has the only NLR/NLR-ID tandem with the NLR (and not the NLR-ID) being a member of MIC1

Generally, MIC1 NLR-IDs were paired with NLRs without an ID from outside MIC1 (Fig. 3, Additional file 4). When we mapped the location of tandems on the NLR phylogeny (Fig. 3; Additional file 4), we observed that MIC1-NLRs from C16 paired exclusively with C7 (32 pairs). We further observed that clades C7, C16, and C17 were involved in more than two-thirds of all tandems (43, 32, or 22, respectively, out of 80). This was consistent with the pairing of *RGA5* from C16 to *RGA4*, which is located in C7 (Fig. 3c). Such pairing of NLRs from two clades might indicate that diversity observed in MIC1 originated from the duplication of an ancestral NLR pair which served as a suitable landing pad for new integrations.

### Microsynteny analysis reveals interchromosomal re-arrangements of NLRs and neighboring genes

**Fig. 4:**
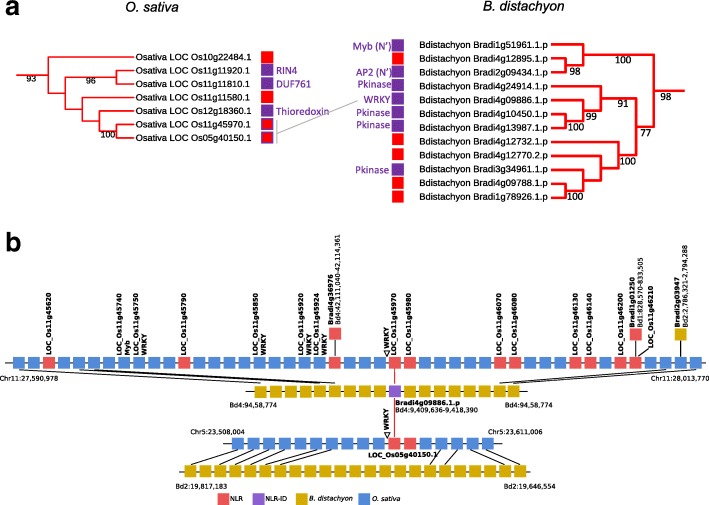
Orthologous NLRs from MIC1 in rice and Brachypodium show NLR gene coupled to generation of new NLR-IDs. **a** Phylogeny of NLRs from MIC1 in rice and Brachypodium based on the NB-ARC domain. *Red boxes* indicate NLRs with IDs. The links between trees highlight orthologous NLRs between rice and Brachypodium. **b** Microsynteny analyses between rice and Brachypodium. *Blue boxes* and *ochre boxes* represent syntenic genes in rice and Brachypodium, respectively. *Red boxes* indicate NLRs, *purple boxes* indicate NLR-IDs

We observed that NLRs from MIC1 were found on different chromosomes across and within species. We analyzed genomic locations of MIC1 NLR-IDs in *O. sativa* and *B. distachyon* (Fig. 4) as these species have highly contiguous genomes. We found syntenic NLR-IDs from MIC1 on chromosome 11 in *O. sativa* and chromosome 4 in *B. distachyon.* These chromosomes contain known syntenic blocks [24], suggesting an ancient origin of the locus that was present in the common ancestor of these grasses. We identified an NLR-ID in *B. distachyon* (Bradi4g09886) with significant hits to two NLRs without an annotated ID in *O. sativa*, one at a syntenic position (LOC_Os11g45970) and the other at a non-syntenic position (LOC_Os05g40150) (Fig. 4a), suggesting inter-chromosomal duplication of NLR pair in rice. Our manual curation (BLASTX of 10-kb region downstream of NLRs against the nr database followed by a Pfam search) showed that these two *O. sativa* loci have an unannotated WRKY domain, similar to Bradi4g09886, downstream of the gene models (Fig. 4b, Additional file 5), which could be due to either gene fission or incomplete annotation.

Furthermore, another MIC1 NLR-ID from *B. distachyon* (Bradi2g09434) has a 1:1 orthologous gene in *O. sativa* (LOC_Os10g22484) that is a non-fused NLR (Fig. 4a, Additional file 5). In both cases, NLR-IDs from Brachypodium have homologs in non-syntenic regions in rice suggesting inter-chromosomal movement of locus with NLR-ID pair (Fig. 4b, Additional file 5).

Interestingly, the regions surrounding NLR-IDs often contain homologs of IDs, including a WRKY and Myb genes on chromosome 11 (Fig. 4b, Additional file 5). Our observations suggest rapid evolution occurs not only in NLR-IDs themselves, but also in the surrounding regions, leading to “trapping” and duplication of ID homologs and rapid loss of microsynteny.

### Identification of new domain integrations at orthologous NLRs

**Fig. 5:**
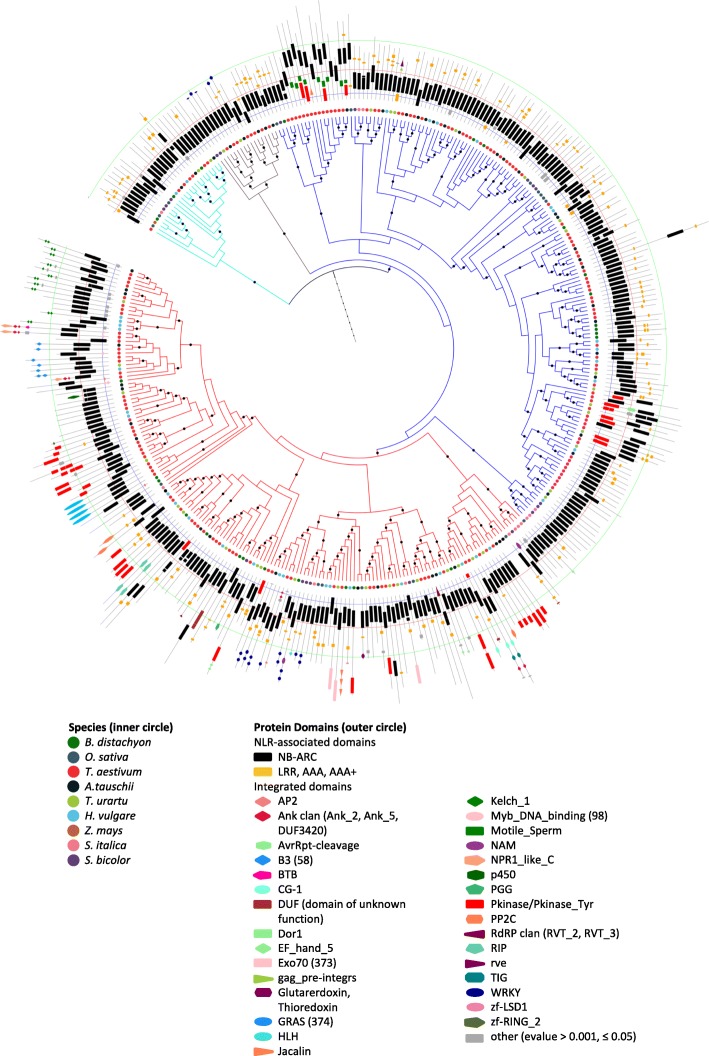
Close-up of MIC1 displaying rapid domain recycling. The *branches* of the hotspot clade, the outer clade, and the ancestral clade are shown in *red*, *blue*, and *cyan*, respectively. *Dots* on the branches indicate a bootstrap support value ≥ 85%. Alongside the tree are cartoons of each protein, annotated with the domain(s) in the position that they appear in the protein (protein backbone, *gray line*; NB-ARC domain, *black rectangle*; LRR and AAA, TIR, and RPW8 domains, *orange rectangles*; other domains in different colors and shapes as indicated in the *key*). For clarity, the domain lengths are shown in the *key* for B3, Exo70, GRAS, and the Myb_DNA_binding domains. E-value cut-off for presence of an ID domain, 0.001; domains with e-value > 0.001 and ≤ 0.05 are shown as *gray rectangles*. E-value cut-off for an LRR, AAA, TIR, or RPW8 domain, 10.0

To further understand the evolution of ID fusions, we examined the proteins from MIC1 and the associated outer and ancestral clades and reconstructed the phylogeny of these clades alone by a maximum likelihood approach (Fig. 5). Each gene was annotated with a figure showing the positions of canonical (NB-ARC and LRR) and non-canonical domains. This representation highlighted differences in the distribution and diversity of the ID domain(s) among the proteins in the ancestral C13, outer C14 and C15, and inner MIC1 clades. The ancestral clade had no ID domains (e-value < 1e-3). The outer clade had two groups of proteins: C15 with kinase domains at their N-terminal ends (all from wheat or its progenitors) and C14 with C-terminal WRKY fusions.

Within MIC1, there are several examples of genes that share the same domain at the same position in the protein. This is particularly apparent in the Triticeae. Among such examples are NLR-GRAS, NLR-kinase, and NLR-NPR1 (Fig. 5). This conservation in architecture indicates a common ancestry and selection to maintain a functional fusion. In contrast to these patterns, many closely related NLR proteins within the inner clade have diverse ID domains, where domains are derived from different protein families and primarily exist near the C-terminal end of the NLR. In some closely related genes, including wheat homoeologs, a variable domain resides in a similar position indicating that there is a common integration point in these genes.

Such a precise integration site raised questions about the mechanisms by which domain integration can be achieved and maintained. For example, did these proteins share nucleotide and/or protein sequences that increase the likelihood of integration events?

### MIC1-NLR-IDs share a protein motif at the site of domain integrations

**Fig. 6:**
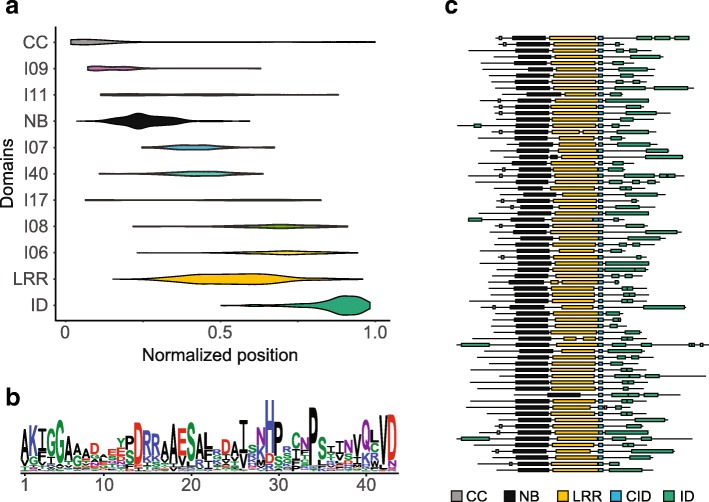
NLR-IDs from MIC1 share a protein motif at the site of domain integrations. **a** Distribution of CC, NB, LRR, and ID domains and motifs identified using MEME on unannotated regions of NLRs within the MIC1 clade with C-terminal ID. For every NLR, the length of the NLR was normalized to 1.0 and the midpoint of identified domains was normalized to protein length. **b** Sequence logo of CID domain [56]. **c** Domain structure of 70 NLR-IDs within the MIC1 clade that contain the CID domain. The CID domain is located immediately upstream of the site of integration

In order to identify shared sequences that might answer the questions above, we searched for protein motifs that were enriched in MIC1. For every protein, we extracted all regions without a domain annotation from InterProScan. Motif prediction using MEME found seven motifs (I06, I07, I08, I09, I11, I17, and I40) that were saturated within MIC1 (Additional file  6). Two motifs, I09 and I11, were associated with the region between the coiled-coil (CC) and NB-ARC domains, whereas I07, I40, I17, I08, and I06 were associated with the LRR region and/or between the LRR and ID (Fig. 6a, C-terminal ID). Motif I09 was widespread, with 80 of 159 proteins in MIC1 harboring this motif, whereas motif I11 occurred in 63 proteins in MIC1. When found together, they were always in tandem (I09-I11) and were located upstream of the NB domain. Motifs I09 and I11 could be regions of the subfamily of CCs and/or NBs found in proteins within MIC1 that were not annotated by InterProScan.

For the group of motifs located between the NB and ID domains, we found that I07, I40, I17, and I08 were LRR motifs trained on regions that were not recognized by InterProScan analysis. These could therefore be excluded from further analysis. In contrast, I06 was a motif specifically associated with NLRs in MIC1 and was located immediately upstream of the integrated domains. Based on its conservation and association with IDs, we designated this domain the CID domain. We developed a Hidden Markov Model trained on the CID domain (Additional files 7 and 8) and superimposed its presence/absence on the phylogenetic tree of NLRs (Additional file 9). The CID domain was present in the majority of genes (70%) within MIC1, occasionally found (20%) in genes in the outer MIC1 clade, and found in nine genes outside these clades. Alignment of the regions encompassing the CID and ID domains uncovered a clear breakpoint between these domains for the majority of NLRs in MIC1 (Fig. 6c). This suggests that while different domains may integrate within NLRs in MIC1, selection acts to maintain the CID domain before the integration site of IDs.

To determine the specificity of the CID domain to NLRs, we searched for the domain in proteins of the nine grass species under investigation. The domain is highly specific to NLRs, with 337 (98%) of positive hits including NLRs (n = 343). Protein structure prediction using Phyre2 found that the CID domain occurs after the last LRR. The position and conserved residues of the CID domain share some similarity to the capping domain of LRRs [27].

### Duplication of genes encoding IDs followed by translocation of either ID or NLR lead to new NLR-ID formation

Evidence from synteny analysis in rice and *B. distachyon* provided an initial understanding of the movement of NLRs across chromosomes in the formation of NLR-IDs. To further elucidate the mechanisms of NLR-ID formation, we looked for examples of the most recent integration. Polyploid bread wheat (*T. aestivum*), presented an ideal system for these analyses. Wheat has an elevated number of NLRs and NLR-IDs (Fig. 2a), a high incidence of new integrations, and multiple orthologous copies of each gene (A, B, and D). The presence of homoeologs allowed us to trace the origin of the ID as well as the translocation of NLR or ID.

To identify proteins that were most closely related to the donor ID domains in *T. aestivum* NLR-IDs, we constructed phylogenies for eight families that harbor ID domains: AP2/ERF, Exo70, GRAS, Kelch_1, NPR1_like_C, Pkinase, Pkinase_Tyr, and WRKY (Additional files 10 and 11) [28]. In many cases, including kinases and AP2 families, we uncovered multiple independent integrations of donor proteins into different NLR proteins. The majority of domains existed as complete domains within the NLR protein suggesting that they might have retained their original function. Additionally, some of the IDs identified might provide access to existing signaling networks; two transcription factor families, for example, identified ID donor proteins for AP2/ERF and WRKY IDs included proteins already known to be involved in stress and pathogen response [29].

We considered three possible mechanisms of NLR-ID formation: (1) retrotransposition of complementary DNA derived from the parental gene; (2) transposition of the parental gene; and (3) ectopic recombination during which double-stranded DNA breaks are repaired using a non-homologous exogenous parental gene as a template. All three mechanisms have been observed previously in cereal genomes [30] and both retrotransposition and ectopic recombination have been suggested as diversification mechanisms of NLRs [31]. We extracted the coding DNA sequences of IDs for 40 *T. aestivum* NLR-ID genes from MIC1 and aligned them back to the genome (BLASTN, e-value 1e-3). Similar to the NLR portion of the genes, the majority of integrated domains contained introns. Therefore, we conclude that retrotransposition of IDs is unlikely.

**Fig. 7:**
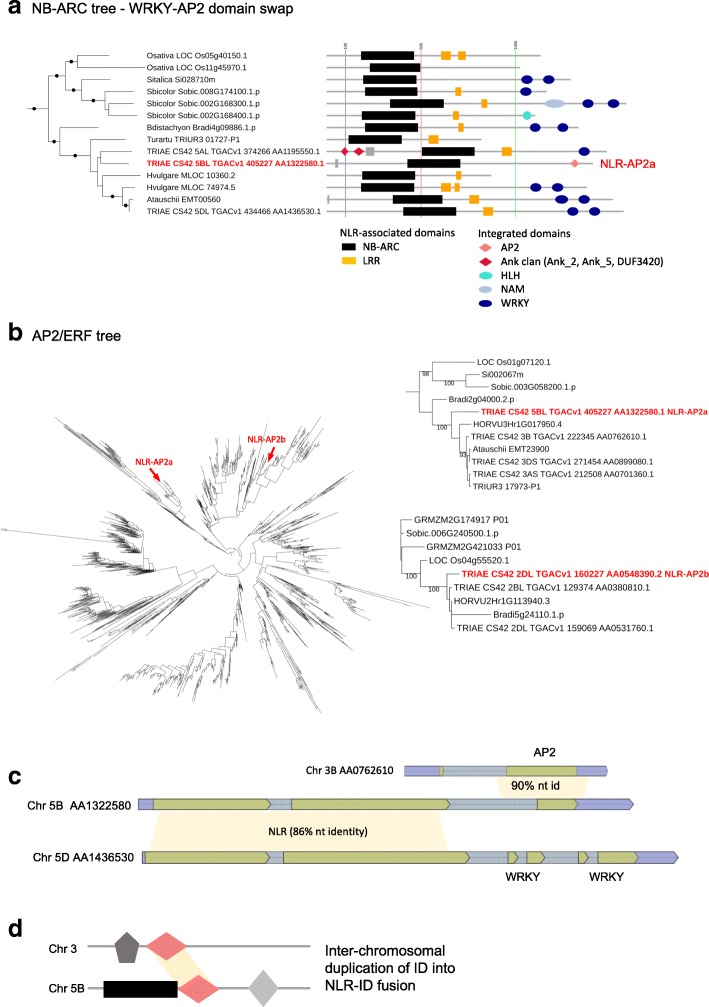
NLR-WRKY/AP2a domain shuffling involved inter-chromosomal copy-and-paste of the AP2 gene. **a** A clade in the phylogeny of NLR-ID proteins from Fig. 5 that includes wheat. A, B and D genome homoeologs from the same genetic position. New integration is evident from homologs with AP2 and a WRKY domains (highlighted by *red boxes*). *Dots* on the tree branches indicate a bootstrap support value ≥ 85%. E-value cut-off for presence of an ID domain, 0.001; a domain with e-value > 0.001 and ≤ 0.05 is shown as a *gray rectangle*. E-value cut-off for an LRR domain, 10.0. **b** An AP2/ERF family tree (*left*) showing two clades that contain an NLR acceptor ID protein (NLR-AP2a and NLR-AP2b, indicated in *red*). The protein sequences of these clades were re-aligned and the trees re-estimated (*right*) to confirm the identity of the donor protein, evident from high bootstrap support values. **c** Protein alignment cartoon of one of the AP2 donor proteins and the acceptor protein, NLR-AP2a. By contrast, the NLR acceptor protein homoeologs contain two WRKY domains in their C-terminal ends (**d**) summary model illustrating the inter-chromosomal duplication of an ID domain and subsequent movement into an NLR gene

To further understand how exogenous domains become fused to NLRs, we investigated a recent exchange of IDs in NLR-IDs, specifically at most recent integrations of AP2 genes in NLR-WRKY/AP2a (Fig. 7) and MYB/AP2b-NLR clades (Additional file 12). In case of NLR-AP2a, we identified three homoeologous NLRs on chromosomes 5A, 5B, and 5D, respectively, with distinct C-terminal fusions (Fig. 7a). The AP2 domain in the NLR-AP2a on chromosome 5BL replaced a more ancient WRKY domain integration present in wheat NLR homoeologs on 5AL and 5DL as well as in other grasses (Fig. 7a). Therefore, the integration of AP2a occurred after the split of the diploid wheat genome progenitors (<4 million years ago) [32].

The closest homolog of the AP2a ID was located on chromosome 3 and was present in all subgenomes (A, B, and D), suggesting a duplication of the parental ID copy either before or coupled with movement into the NLR (Fig. 7b). By aligning the AP2a nucleotide sequence to its parental genes on chromosome 3 with BLASTN, we observed that integration involved a part of AP2 intron 1 and exon 2, which became fused with the intron 2 of NLR displacing the WRKY gene (Fig. 7c). Since the three parental AP2 genes were intact and there are no additional paralogs on any subgenome, we concluded that the gene must have been copied first into a new location if transposition had been involved. We found no evidence of the residual first exon of AP2 in any wheat subgenome. A recent transposition would also have left a footprint, such as terminal inverted repeats. A BLASTN search of the ID sequence and its surrounding region against itself revealed only a very short repeat (TATAGCTACAG) on each side of ID. The presence of short terminal inverted repeats suggests that if transposition had been involved, it would have been mediated through a poorly characterized class of DNA transposon, such as hAT or PIF/Harbinger. The linker region between the NB-ARC and AP2 domains also contains the 86-bp inverted repeat with no similarity to characterized TEs. Neither the short 11-bp repeat sequence nor the 86-bp inverted repeat were shared with other NLR-IDs. Moreover, the whole 900-bp nucleotide region between the last exon of NLR and the start of ID as well as nucleotide sequence immediately downstream of ID have no similarity (BLASTN, e-3) to any other wheat NLR.

In the second example, an AP2 domain displaced the MYB domain as N-terminal fusion of NLR (Additional file 12). The AP2b gene is evolutionary distinct from AP2a (Fig. 7b), representing an independent fusion of a distant family member. Interestingly, in this example, it was the NLR that moved into new location since the AP2b-NLR is located on chromosome 2DL and its NLR homoeologs were located on chromosome 7AS and, via a known large-scale chromosomal translocation from 7BS [33], on to chromosome 4AL (Additional file 12). Moreover, chromosome 2DL contains a non-fused copy of AP2b gene indicating that, as in the case of AP2a, the parental ID gene was duplicated before integration. While we saw more examples of distinct IDs fused to orthologous NLRs (Fig. 6), this wheat example together with our rice/*B. distachyon* analyses suggested that either the ID or NLR can be translocated to a distinct genomic location to create a new fusion event.

Transposable elements (TEs), such as Helitrons and Pack-MULEs, are known to capture gene fragments and therefore can lead to new gene fusions [34, 35]. We searched for the presence of Helitrons and MULEs near NLRs and looked for any prevalence of these elements in MIC1 NLRs. For Helitrons, we used HelitronScanner [36] to scan the full genomes of *O. sativa* and *B. distachyon* and the NLR-containing scaffolds of *T. aestivum*. We found no increased prevalence of Helitrons next to MIC1 NLRs compared to other clades (Additional file 13) [28]. For analyses, we took advantage of RiTE-db, a well annotated database of TEs in rice, which includes over 200,000 characterized repetitive elements [37]. We blasted genomic sequences of *O. sativa* NLRs against RiTE-db (BLASTN, e-10) and observed that while MULEs from different families are present in 6/7 rice MIC1 NLRs, they are not placed on either side of IDs or NLRs and most of rice NLRs contain similarity to MULEs on either side of the gene [28]. Therefore, while MULEs might play a role in the duplication of NLRs, there is no sufficient evidence to suggest that they drive NLR-ID formation. We also searched the MIC1-specific CID protein motif against TRansposable Elements Platform (TREP) database [38] and found no significant hits.

Overall, our analysis suggests that the integration of exogenous domains into NLRs follows duplication of IDs in addition to an interchromosomal gene translocation mechanism. Although we did not find clear TE-associated motifs in MIC1 NLR-IDs, such a “copy-and-paste” mechanism can involve TEs with more elusive footprints, such as hAT or previously uncharacterized elements. It is also likely that if TEs are involved, the footprints are rapidly eroded and intra-species analyses are needed to catch the signal from recent integrations Alternatively, NLR-IDs could be generated through ectopic recombination. Further analysis of the most recent intergrations might help to pinpoint the mechanism of NLR-ID formation.

## Conclusions

**Fig. 8:**
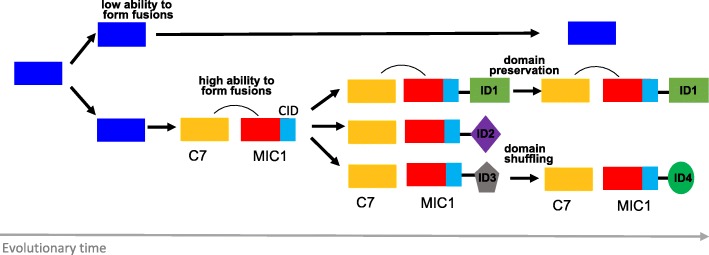
Evolutionary model of NLR-ID hotspot formation and diversification. An ancestral protein underwent duplication to form the outer clade of proteins (*blue*). An ancestral pair between MIC1 and C7 formed as well as the CID LRR-cap motif evolved to be MIC1-specific. Together, this enabled MIC1 to be highly amenable to new gene fusions both on genetic and protein regulatory levels. Duplications of the MIC1-C7 pair created new landing pads for IDs. Some proteins have maintained the same domain (e.g. ID1) but other proteins have undergone further diversification through the exchange of the ID domain (e.g. ID3 and ID4)

We have investigated the formation of NLR-IDs in grasses and demonstrated that while many NLR clades are capable of new domain integrations, the distribution of NLR-IDs is uneven across the NLR phylogeny. While some clades which are rich in NLR-IDs represent the proliferation of single ancient domain integrations, one dominant clade MIC1 harbors the most diverse NLR fusions. MIC1 includes several known NLR-IDs, such as rice *RGA5* and *Pi-ta*, as well as barley *Rpg5*. The NLRs in MIC1 are often genetically linked to another NLR originating from a distinct clade, C7, which includes *RGA4*, a known partner of *RGA5*. Previous studies of RGA5 and RGA4 showed that the two proteins form a complex in which the NLR-ID serves as a pathogen sensor and its NLR partner provides a signaling platform [10]. We hypothesize that the ancient pairing of NLRs from MIC1 and C7 might have enabled the MIC1 genes to be more amenable to fusions (Fig. 8). An NLR that can tolerate new domain integrations provided an evolutionary benefit to the plant diversifying sensors for pathogen effectors, while the C7 partner took over the signaling role.

Strikingly, MIC1 NLRs acquire IDs at a similar position within the protein, located right after the MIC1-specific CID motif. We showed that the CID domain has similarity to an LRR-cap, a structural motif that is often found at the end of the leucine rich repeats [27]. The specificity of CID sequence to MIC1 NLRs suggests that this motif might have enabled MIC1 NLRs to tolerate new fusions (Fig. 8), either playing a regulatory role in NLR-ID activation or directing the integration mechanism itself. Currently, we have found no evidence that the CID motif is associated with TEs.

In MIC1, new NLR formation is an active mechanism that involves inter-chromosomal gene movement. Our synteny analyses between rice and *B. distachyon* documented the movement of NLRs as well as the rapid loss of synteny in regions surrounding NLR-IDs. Interestingly, homologs of IDs were often present in the same genomic regions as MIC1 NLR-IDs. Although we cannot exclude transposition as a mechanism for NLR-ID formation, we have not observed any known TE or TE-associated inverted repeats with specific association to MIC1 genes. Unequal recombination across MIC1 genes themselves or among the surrounding genes might facilitate NLR-ID formation.

How the plant innate immune system acquires new pathogen recognition specificities remains a key question in plant–pathogen interactions. Its answer is closely linked to the evolution of plant immune receptors and their diversification. The processes underpinning genome evolution often include domain duplication, fission, and fusion [39], which have recently been implicated in NLR evolution [7–9, 40]. One of the most advantageous pathways to recognize multiple pathogens is by guarding common plant proteins that are targeted by multiple, if not all, pathogens. However, such mechanism involves self-recognition and can quickly lead to auto-immunity [41, 42]. Genetic linkage of NLRs and their binding partners into NLR-IDs and NLR-NLR pairs can prevent allele shuffling and autoimmunity. This also enables coordinated transcription and translation beneficial for controlling protein stoichiometry and co-evolution. Therefore, a clade of NLRs which has gained the capacity to “integrate” new domains presents an evolutionary advantage.

In the future, the availability of higher quality genome assemblies as well as multiple genomes for each species will allow more detailed analyses of syntenic gene clusters and will identify the precise locations of DNA breakpoints that lead to NLR-ID formation. Combining long molecule RenSeq [43] with population genetics analyses will allow us to estimate how rapidly new gene fusions are formed within populations and how fast the selection of advantageous combinations occurs in nature.

There is an urgent need for new genetic sources of resistance for future sustainable crop production [44, 45]. Our identification of NLRs that are highly amenable to the integration of exogenous domains can be efficiently exploited for advancing the understanding of how new immune receptor specificities are formed and provide new avenues to generate novel synthetic fusions.

## Methods

### Identification of NLRs and NLR-IDs in plant genomes

NLR plant immune receptors were identified in nine monocot species by the presence of the common NB-ARC domain (Pfam PF00931) as described previously [9], except that updated genome datasets for *T. aestivum* (TGAC v1), *A. tauschii* genomes (ASM34733v1), and barley [46] were downloaded from EnsemblPlants and analyzed with the same pipeline used previously [9]. This analysis included the identification of proteins with “integrated” domains (Additional file 14) All scripts are available from https://github.com/krasileva-group/plant_rgenes, script versions used in this study include K-parse_Pfam_domains_NLR-fusions-v2.4.pl and K-parse_Pfam_domains_v3.1.pl.

To ensure comparable comparisons of NLRs and NLR-IDs across species, the quality of the protein annotation data was assessed by the core gene content expected to be present in the plant Embryophyta lineage using BUSCO [47] with the embryophyta_odb9 lineage file.

### Phylogenetic analysis

The NB-ARC Pfam model PF00931 was extended to include the ARC2 subdomain which is present in plant NLRs but absent in the default Pfam model. To build the model of NB-ARC1-ARC2, eight PF00931 seed proteins (SwissProt identifiers: APAF_HUMAN, LOV1A_ARATH, K4BY49_SOLLC, RPM1_ARATH, R13L4_ARATH, RPS2_ARATH, DRL24_ARATH, DRL15_ARATH) were aligned using PRANK [48] and the HMM was built from this alignment with HMMER3 HMMBUILD [49], using default parameters for both programs (Additional files 15 and 16).

Amino acid sequences encoding all NB-ARC proteins identified in nine grass species were aligned to the NB-ARC1-ARC2 HMM using the HMMER3 HMMALIGN program (version 3.1b2) [49]. The resulting alignment of the NB-ARC1-ARC2 domain was converted to fasta format using HMMER ESL-REFORMAT. Any gap columns in the alignment of target proteins with the HMM were removed. Sequences with < 70% coverage across the alignment were removed from the dataset to reduce false placement in the tree of sequences with insufficient coverage across the domain. The longest sequence for each gene out of the available set of splice versions was used for phylogenetic analysis. In addition, 38 proteins with characterized and known functions in pathogen defense from the literature were also included; the list of genes was based on a curated R-gene dataset by Sanseverino et al., 2012 (http://prgdb.crg.eu) {Sanseverino, 2013 #37}. The final alignment that was used for phylogeny is available at figshare [28].

Phylogenetic analysis was carried out using the MPI version of RAxML (v8.2.9) [50] with the following method parameters set: -f a, -x 12345, -p 12345, -# 100, -m PROTCATJTT. The tree contained 4184 sequences and 338 columns, took 67 h to generate, and required 17 GB RAM. Separate trees for each species were also prepared using the same methods (Additional file 3). The overall species phylogeny was constructed using NCBI taxon identification numbers at phyloT (phylot.biobyte.de).

All trees were mid-point rooted and visualized using the Interactive Tree of Life (iToL) tool [51] The trees are openly available at iToL in interactive mode (search for KrasilevaGroup or see links in “Data Availability” below). Annotation files were prepared for displaying the presence of ID domains in the proteins, depicting species gene identifiers by color, and visualizing the location of individual domains within the protein backbone. An ID domain was defined as being any domain, except for NB-ARC itself, LRR, AAA, TIR, and RPW8, which are often associated with NB-ARC-containing proteins. NLR clade membership (Additional file 2) was defined based on average BRL (> 1.4) and bootstrap support (>80%). Clades 16 and 24 exhibited low internal bootstrap support and were defined based on differentiation from other clades with strong bootstrap support.

To identify donor genes for the ID domains of the NLR-ID proteins, phylogenetic trees for eight donor gene families (AP2, Exo70, GRAS, Kelch_1, NPR1_like_C, Pkinase, Pkinase_Tyr, and WRKY) were produced by the methods described above, except that the species chosen were *A. thaliana*, *M. truncatula*, *B. distachyon*, and *T. aestivum*, except for the ERF family for which *A. thaliana*, *M. truncatula*, and all nine monocot species described above for the NLR family were included. The following protein annotation files were used: *A. thaliana* (TAIR10_pep_20101214_updated (TAIR10)), *B. distachyon* (Bdistachyon_314_v3.1.protein.fa (Phytozome, version 12)), *M. truncatula* (Mtruncatula_285_Mt4.0v1.protein.fa (Phytozome, version 12)), and *T. aestivum* (TGAC_v1 protein annotation as described above (EnsemblPlants website)). The HMMs used for each family were taken from the Pfam-A database (Release30), except for the model for the AP2/ERF domain, which was created from an alignment with PRANK of *A. thaliana* and rice ERF proteins. Protein sequence alignments used for the trees are available at figshare [28] and the trees are available for download from the group’s project in iToL.

### Identification of protein motifs

NLRs within MIC1 were annotated for known domains using InterProScan (v5.20-59.0). Domains were annotated and undefined regions within NLRs were extracted using the QKdomain pipeline (https://github.com/matthewmoscou/QKdomain). All undefined regions were required to be at least 20 amino acids long. MEME (v4.11.2) was used for motif prediction on the extracted regions [52]. FIMO was used to identify motifs in the entire set of NLRs from diverse grass species [52]. Visualization of the presence/absence of motifs was performed using iToL [51]. Multiple sequence alignments were performed using MUSCLE (v3.8.31) [53]. HMMER3 (v3.1b1) HMMBUILD was used to train Hidden Markov Models on conserved sequences and HMMSEARCH was used to search the entire NLR dataset, using default parameters [49]. The complete pipeline, including scripts and datasets, is available from the Github repository NLR-ID_motif (https://github.com/matthewmoscou/NLR-ID_motif).

### Detection of paired NLRs

Gene coordinates were obtained from the Phytozome (V10) GFF annotation files for *B. distachyon* (283_v2.1), *O. sativa* (204_v7.0), *S. bicolor* (255_v2.1), *S. italica* (164_v2.1), and *Z. mays* (284_6a). Gene annotation for *T. aestivum* was obtained from Earlham Institute (http://opendata.earlham.ac.uk) [25] and for barley from Mascher et al. [46]. The gene annotations of *A. tauschii* (ASM34733v1.33) and *T. Urartu* (ASM34745v1.33) were obtained from Ensembl Plants.

All NLR genes (both complete and partial across the NLR domain) were tested for the presence of paired NLRs (NLR1 upstream of NLR2, NLR1 in reverse orientation, NLR2 in forward orientation, no other gene and a maximum distance of 15 kbp between the NLRs). The paired NLR search was performed with tandem.py (https://github.com/krasileva-group/tandem) and the results were displayed using Circos [54]. Statistical significance was calculated with Fisher’s exact test (as implemented in scipy).

### Searching for TE-associated motifs

The CID motif was aligned across all MIC1 NLRs and converted into an HMM using HMMER3 [49] (Additional file 8). The resulting HMM was scanned against the TREP database [38] (http://botserv2.uzh.ch/kelldata/trep-db/index.html).

Helitrons were identified using HelitronScanner [36] with default parameters against the full genomes of *O. sativa*, *B. distachyon*, and the NLR-containing scaffolds of *T. aestivum*. We then converted the coordinates of Helitrons predicted by HelitronScanner into bed format and compared them against the coordinates of all NLR genes from these species using bedtools intersect with default parameters [55]. Overlaps between NLRs and Helitrons were plotted in R using the ggplot2 library.

MULE elements are well characterized only in the *O. sativa* genome and are part of the RiTE database [37]. We compared genomic sequences of all *O. sativa* NLRs against all TEs in RiTE using BLASTN (e-10, culling_limit 1) [28]. We analyzed all TEs and specifically MULEs matching NLRs and plotted results in R using the ggplot2 library.

## Additional files

Additional file 1: Quality assessment of the gene datasets used for phylogenetic analysis: N50 and size of the corresponding genome assembly, number of NLR genes with incomplete coverage (< 70%) across the NB-ARC1_ARC2 domain and BUSCO analysis of the primary genes in the protein annotation. (XLSX 33 kb)

Additional file 2: Clade memberships of all NLRs present in the tree in Fig. 1 and corresponding integrated domains present in NLR-IDs (evalue <= 0.05). (TSV 157 kb)

Additional file 3: Maximum likelihood phylogeny based on the NB-ARC domain of all NLRs and NLR-IDs for each of the nine grass species under study. (A) *S. italica*, (B) *S. bicolor*, (C) *Z. mays*, (D) *O. sativa*, (E) *B. distachyon*, (F) *H. vulgare*, (G) *A. tauschii*, (H) *T. aestivum*, and (I) *T. urartu*. Proteins with integrated domains are represented by red squares. Clades of interest are colored as following: MIC1 (red); outgroup clades C14–15 (blue); and ancestral clade C13 (cyan). (PPTX 7164 kb)

Additional file 4: Locations, gene identifiers, and tandem classifications for all discovered tandem NLRs in eight grass species. (XLSX 40 kb)

Additional file 5: Manual curation of the genomic regions surrounding MIC1 genes in Brachypodium and rice. (A) MIC1 NLR-IDs and surrounding genes. (B) Additional microsynteny analyses between rice and *B. distachyon*. (PPTX 150 kb)

Additional file 6: Motifs identified using MEME that are associated with MIC1 clade. (PDF 866 kb)

Additional file 7: Alignment file used to generate HMM for the CID domain. (FA 4 kb)

Additional file 8: The HMM for the CID domain. (HMM 18 kb)

Additional file 9: Presence/absence of motifs (marked as black dots) relative to the NB phylogenetic tree. (A) I06, (B) I09, and (C) I11. (PDF 2852 kb)

Additional file 10: Maximum likelihood phylogeny for eight gene families containing proteins with ID domains that were used to identify potential donor genes within each family for *T. aestivum* NLR-ID genes from the MIC1 clade. The gene identifiers highlighted in red are the acceptor genes containing an NB-ARC domain. (A) AP2/ERF family, (B) Exo70 family, (C) GRAS family, (D) Kelch_1 family, (E) NPR1_like_C, (F) Pkinase, (G) Pkinase_Tyr, and (H) WRKY. (PPTX 9868 kb)

Additional file 11: Potential donor - NLR acceptor gene sets, as observed from phylogenetic trees of the donor ID genes. High bootstrap support (> 85) was used to determine likely donor ID and acceptor NLR-ID gene clades. (XLSX 28 kb)

Additional file 12: The AP2b/MYB-NLR domain shuffling includes duplication of AP2 gene and inter-chromosomal transfer of NLR. (PDF 2232 kb)

Additional file 13: Bar plot of number of NLRs in each clade and percent of NLRs from each clade that overlap with predicted Helitrons. (PDF 10 kb)

Additional file 14: All NLR-IDs found in nine grasses (e-value at relaxed cut-off < 0.05), including the genes that could not be included in the tree. (TSV 48 kb)

Additional file 15: The alignment of NB, ARC1, and ARC2 used to train the HMM for NLR proteins used for phylogenetic analysis. (FASTA 3 kb)

Additional file 16: The HMM trained from alignment of NB, ARC1, and ARC2 for NLR proteins used for phylogenetic analysis. (HMM 154 kb)
