# Supplementary material for: Dominant integration locus drives continuous diversification of plant immune receptors with exogenous domain fusions
Source: Genome Biol. 2018 Feb 19;19:23. doi: 10.1186/s13059-018-1392-6 (PMC5819176; doi:10.1186/s13059-018-1392-6)
Supplement: Supplementary file 3 — Maximum likelihood phylogeny based on the NB-ARC domain of all NLRs and NLR-IDs for each of the nine grass species under study. (A) S. italica, (B) S. bicolor, (C) Z. mays, (D) O. sativa, (E) B. distachyon, (F) H. vulgare, (G) A. tauschii, (H) T. aestivum, and (I) T. urartu. Proteins with integrated domains are represented by red squares. Clades of interest are colored as following: MIC1 (red); outgroup clades C14–15 (blue); and ancestral clade C13 (cyan). (PPTX 7164 kb) [file 13059_2018_1392_MOESM3_ESM.pptx]

## Slide 1
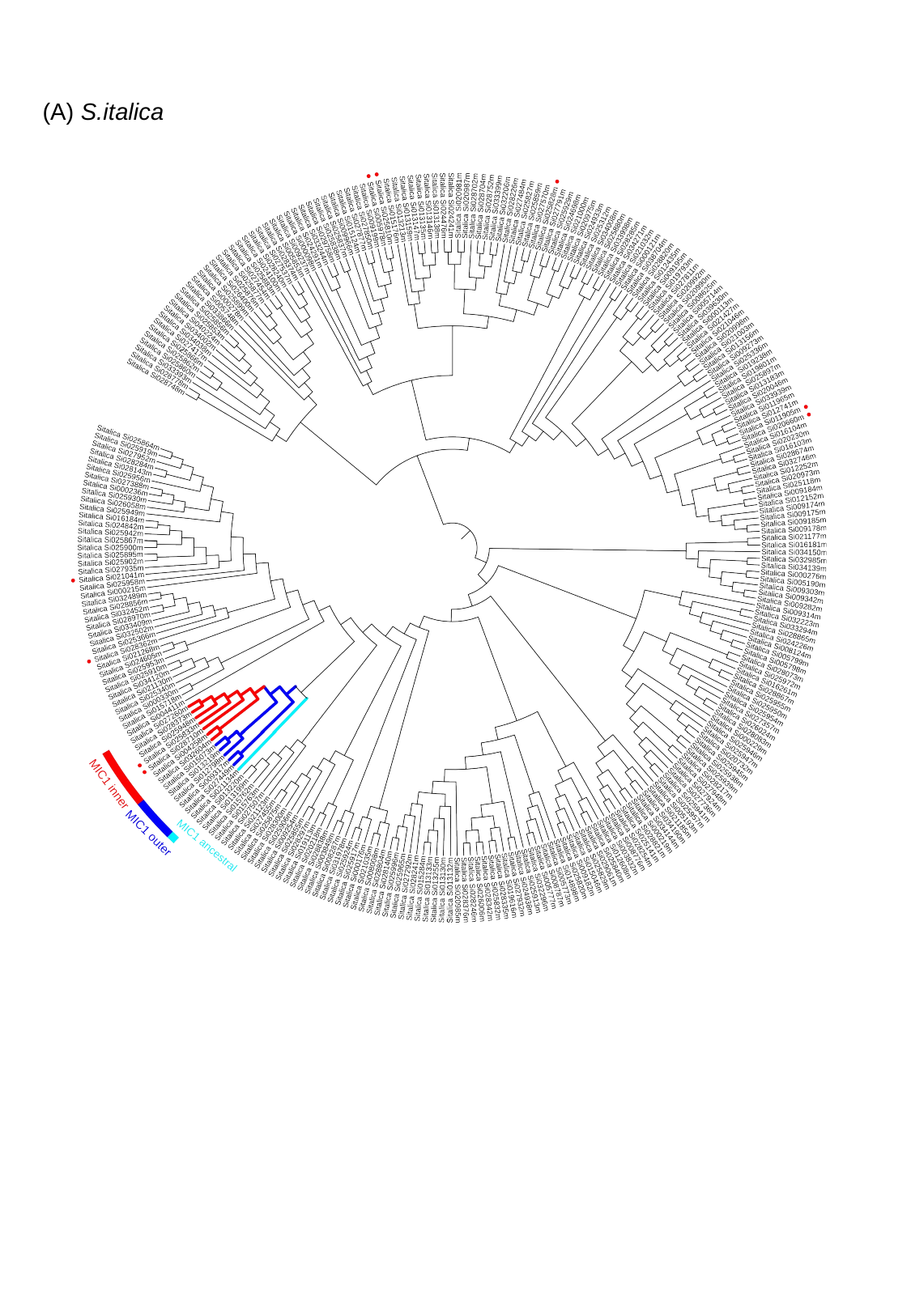

(A) S.italica

## Slide 2
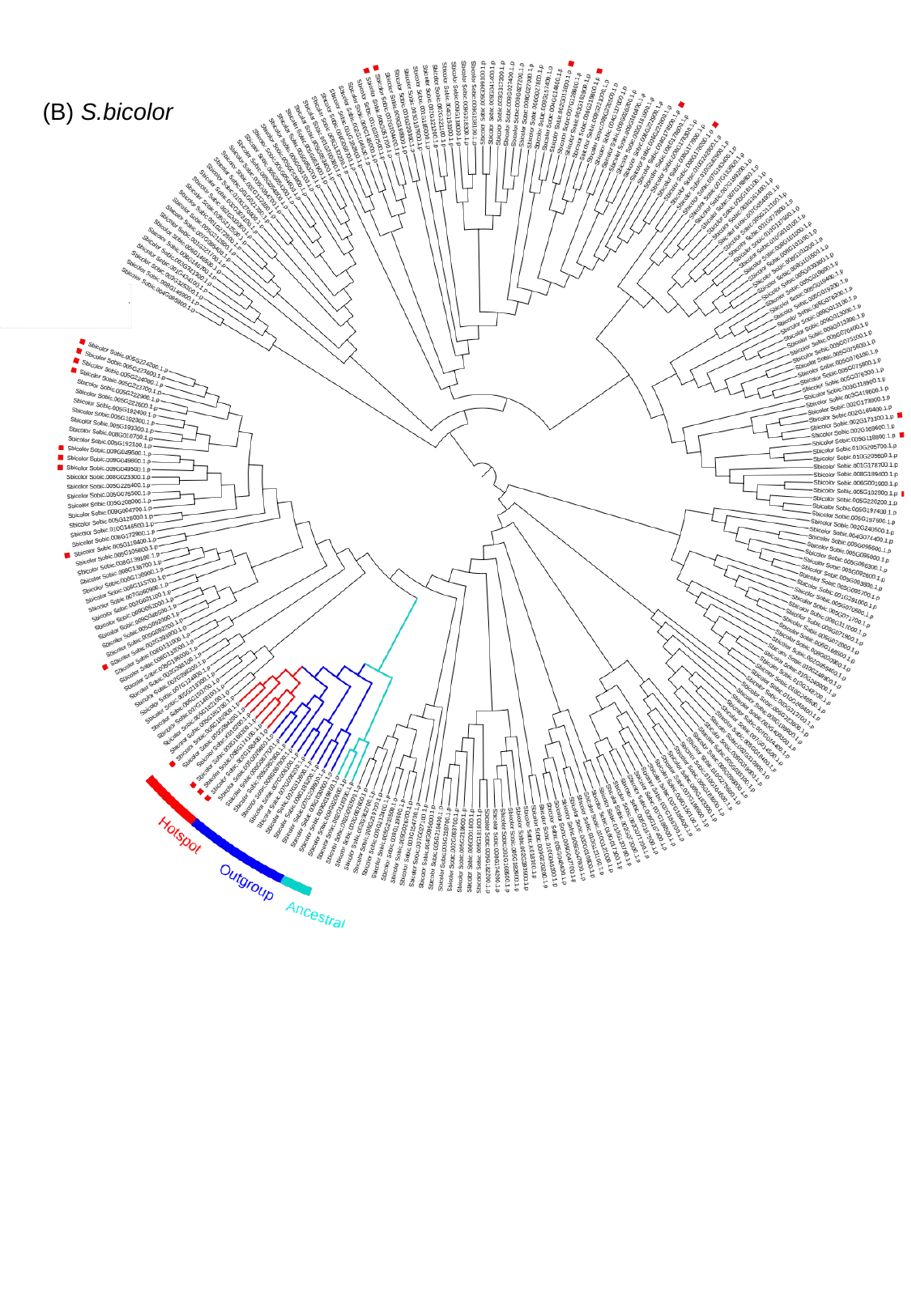

(B) S.bicolor

## Slide 3
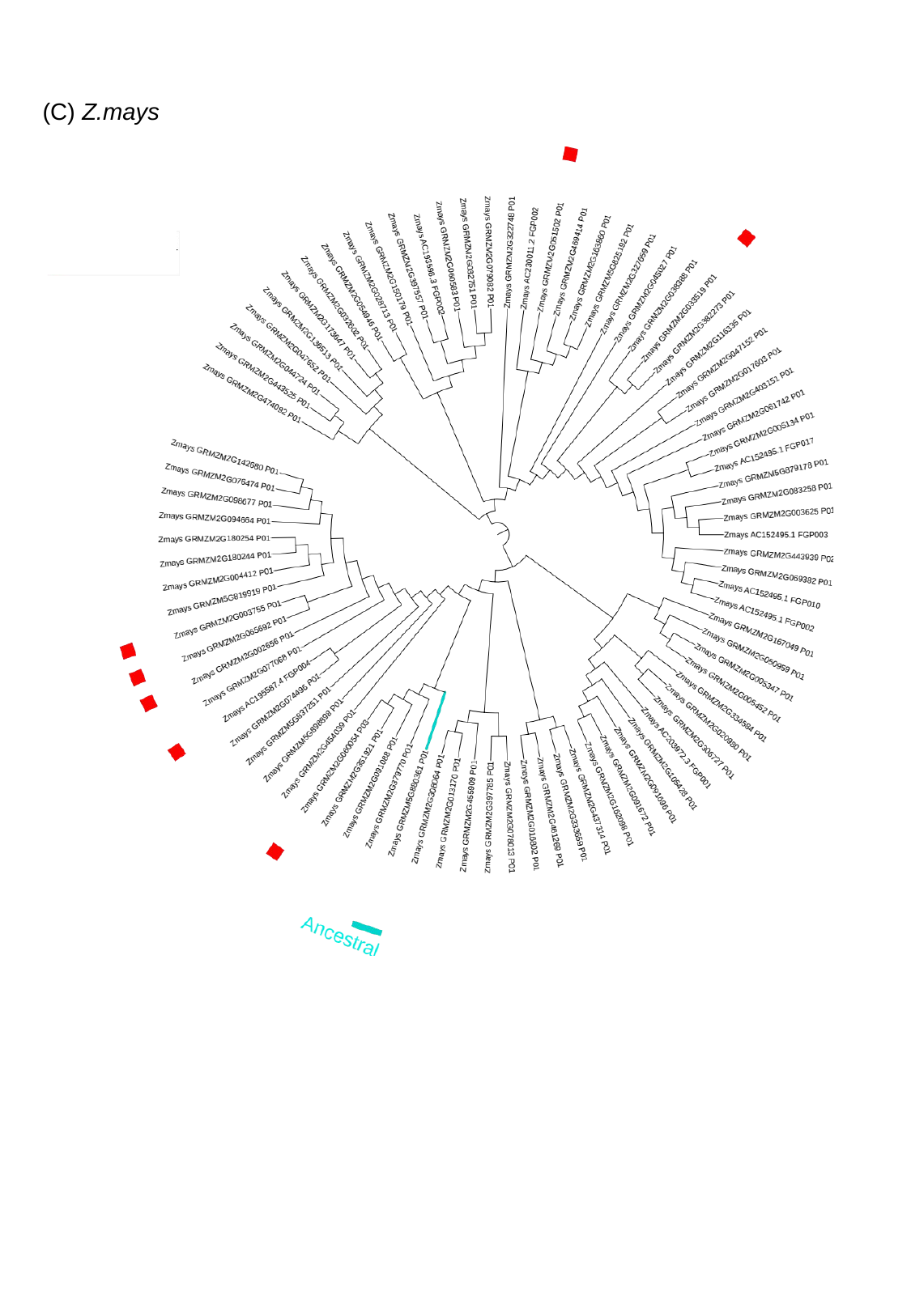

(C) Z.mays

## Slide 4
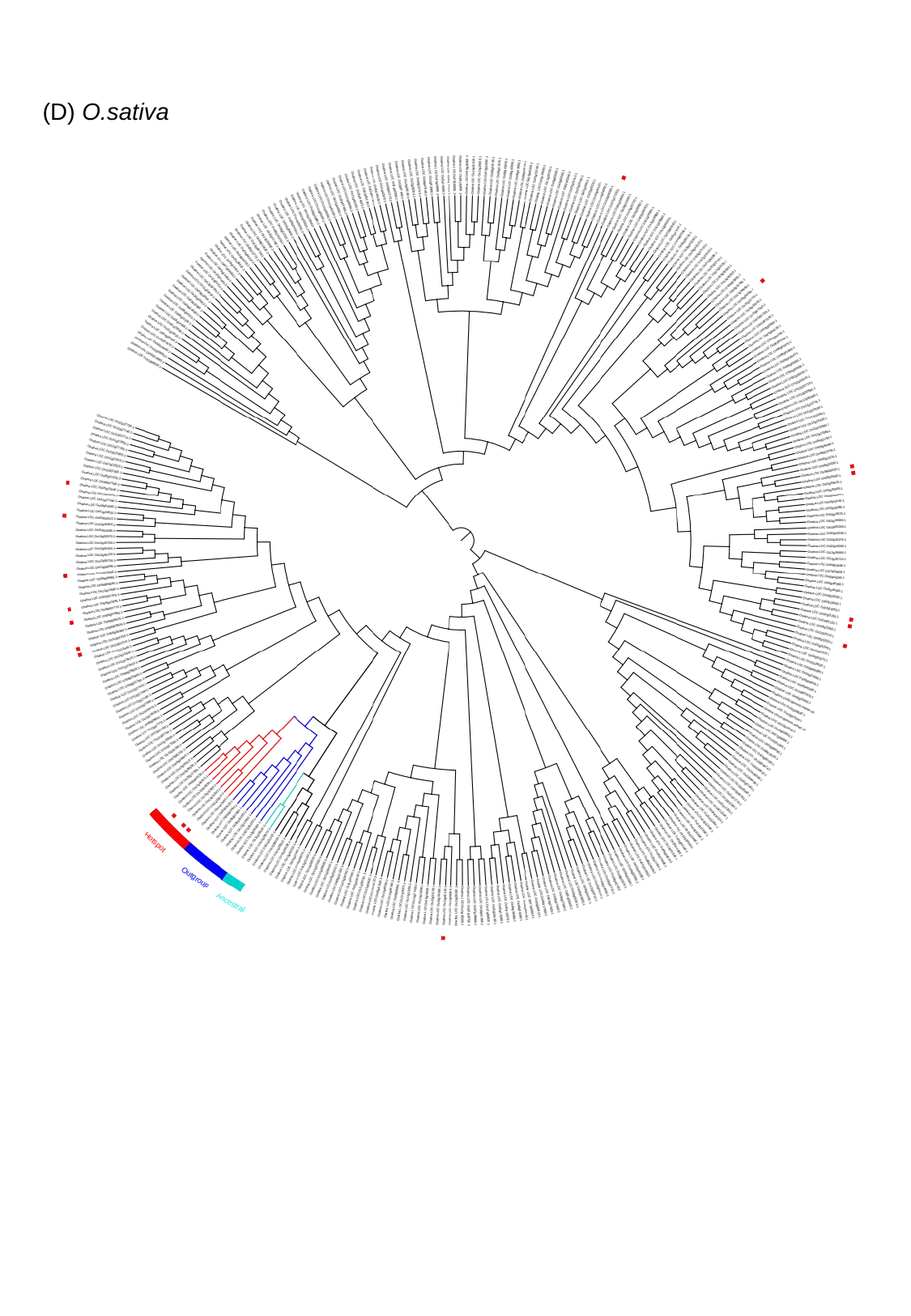

(D) O.sativa

## Slide 5
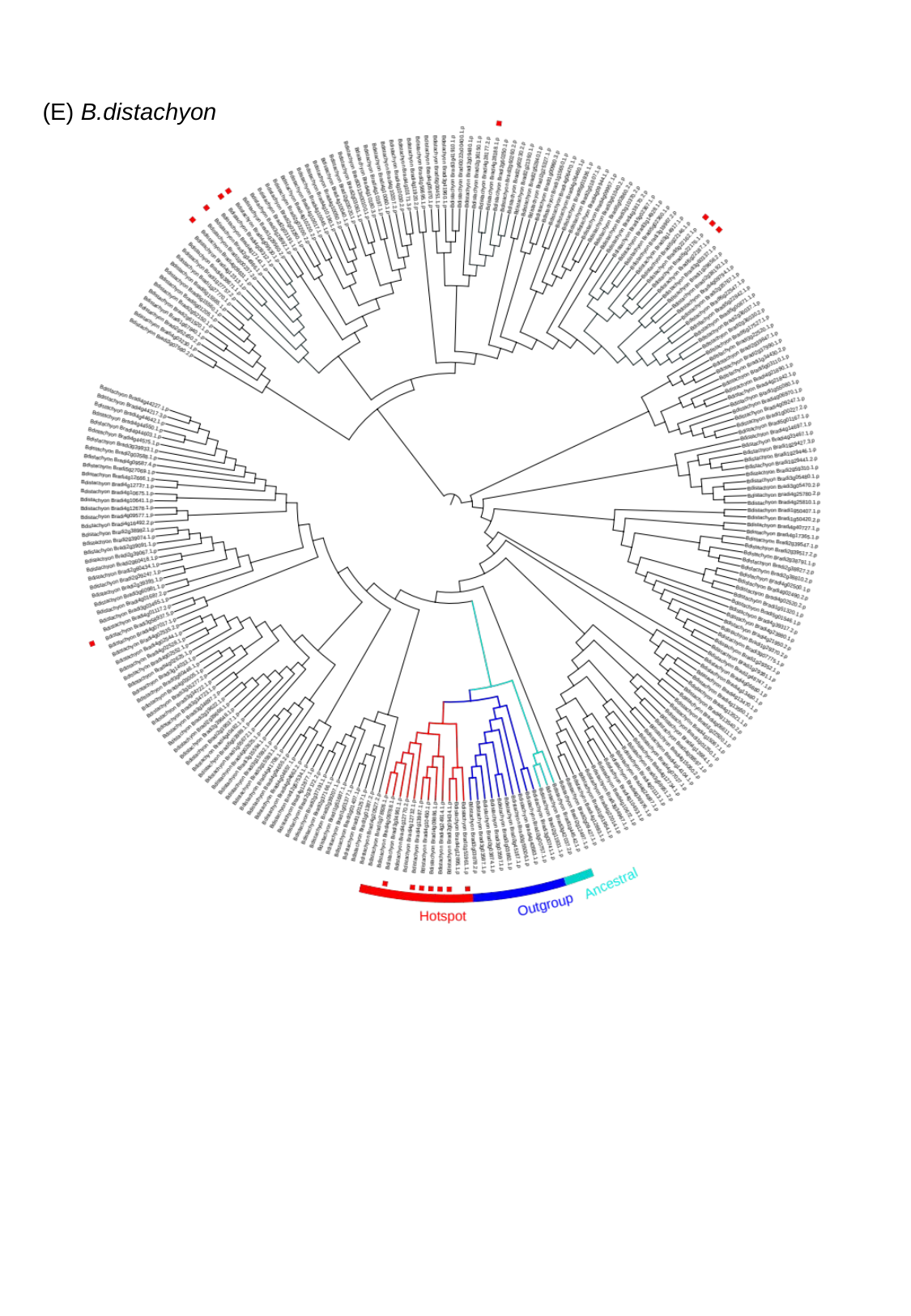

(E) B.distachyon

## Slide 6
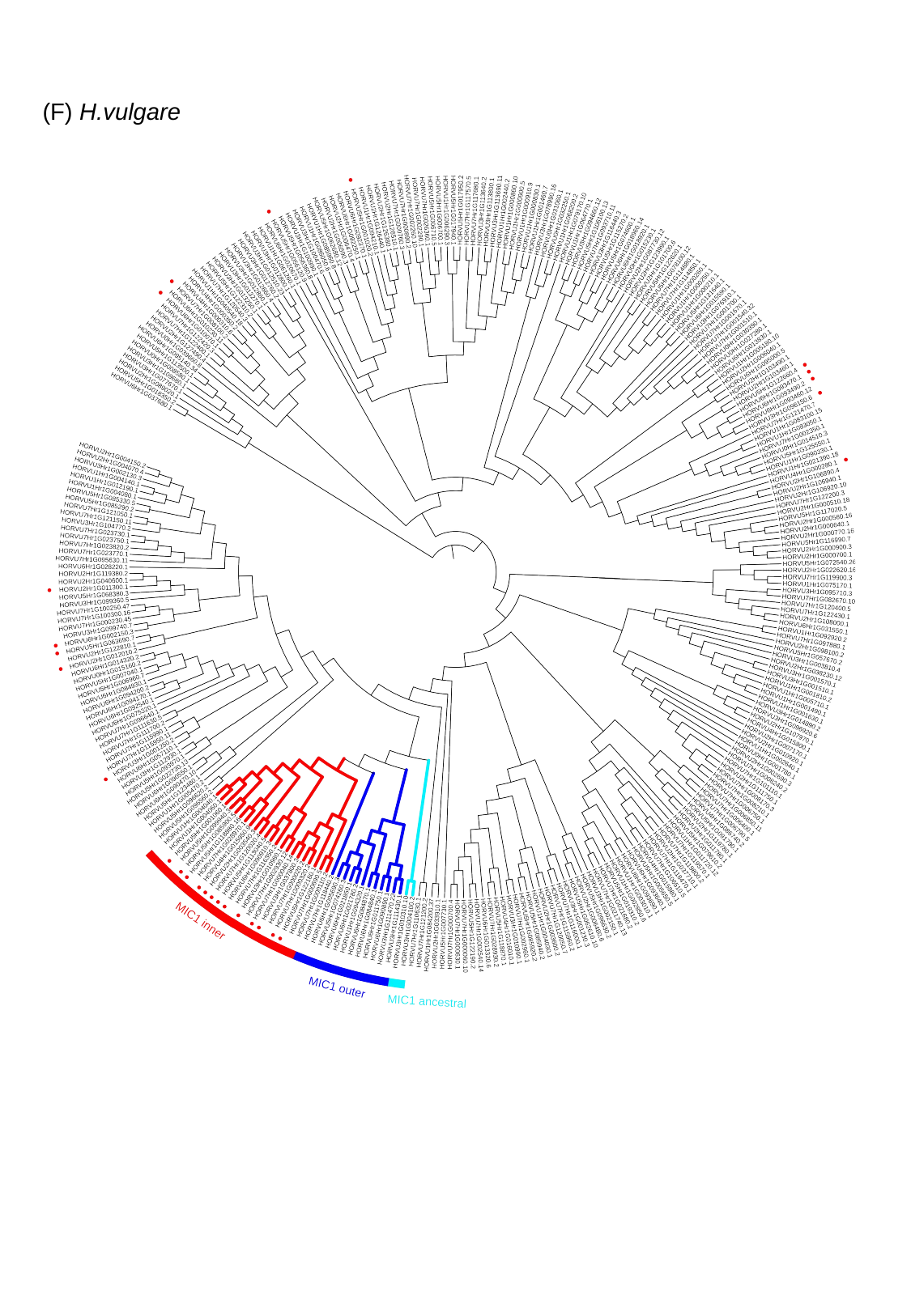

(F) H.vulgare

## Slide 7
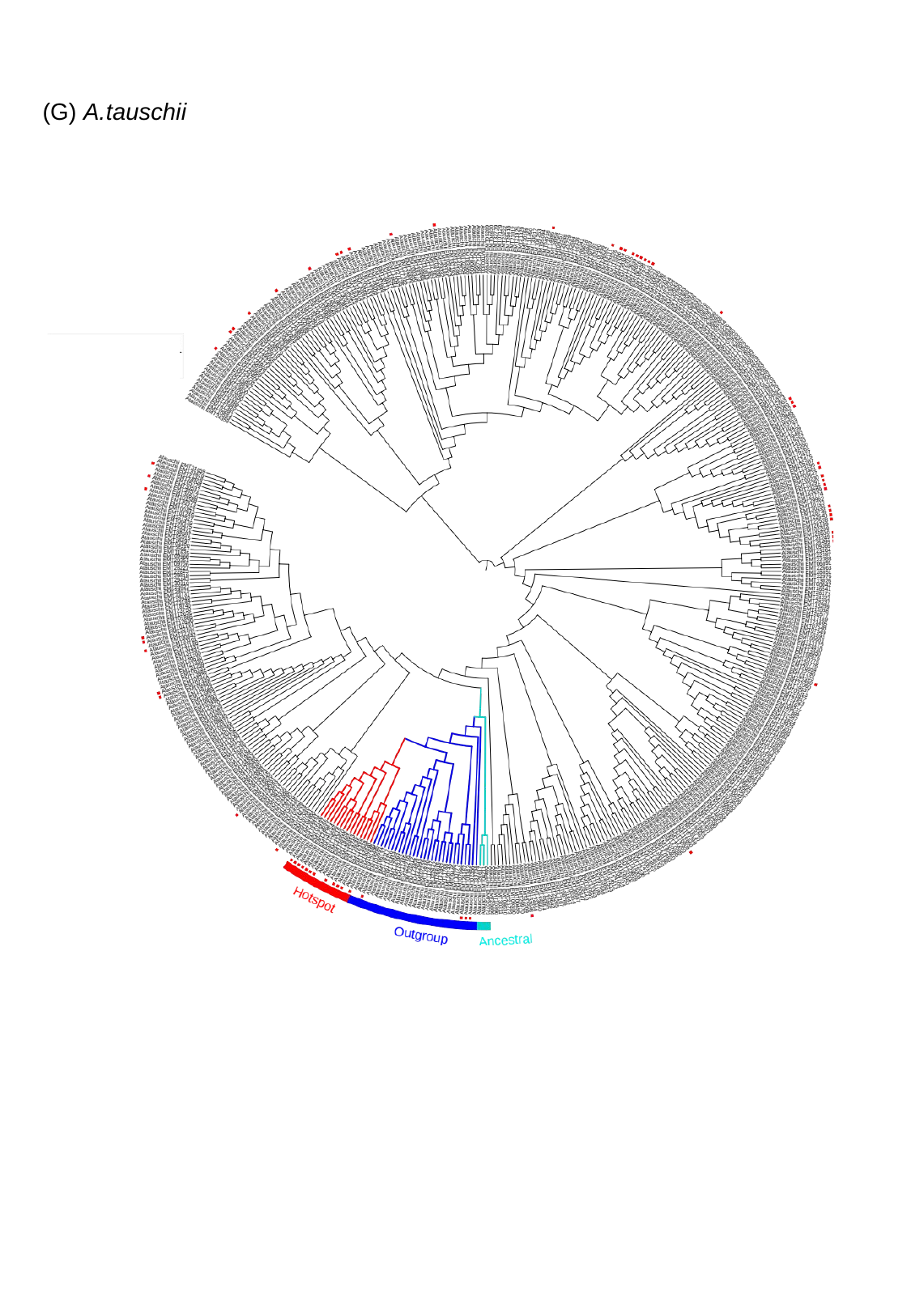

(G) A.tauschii

## Slide 8
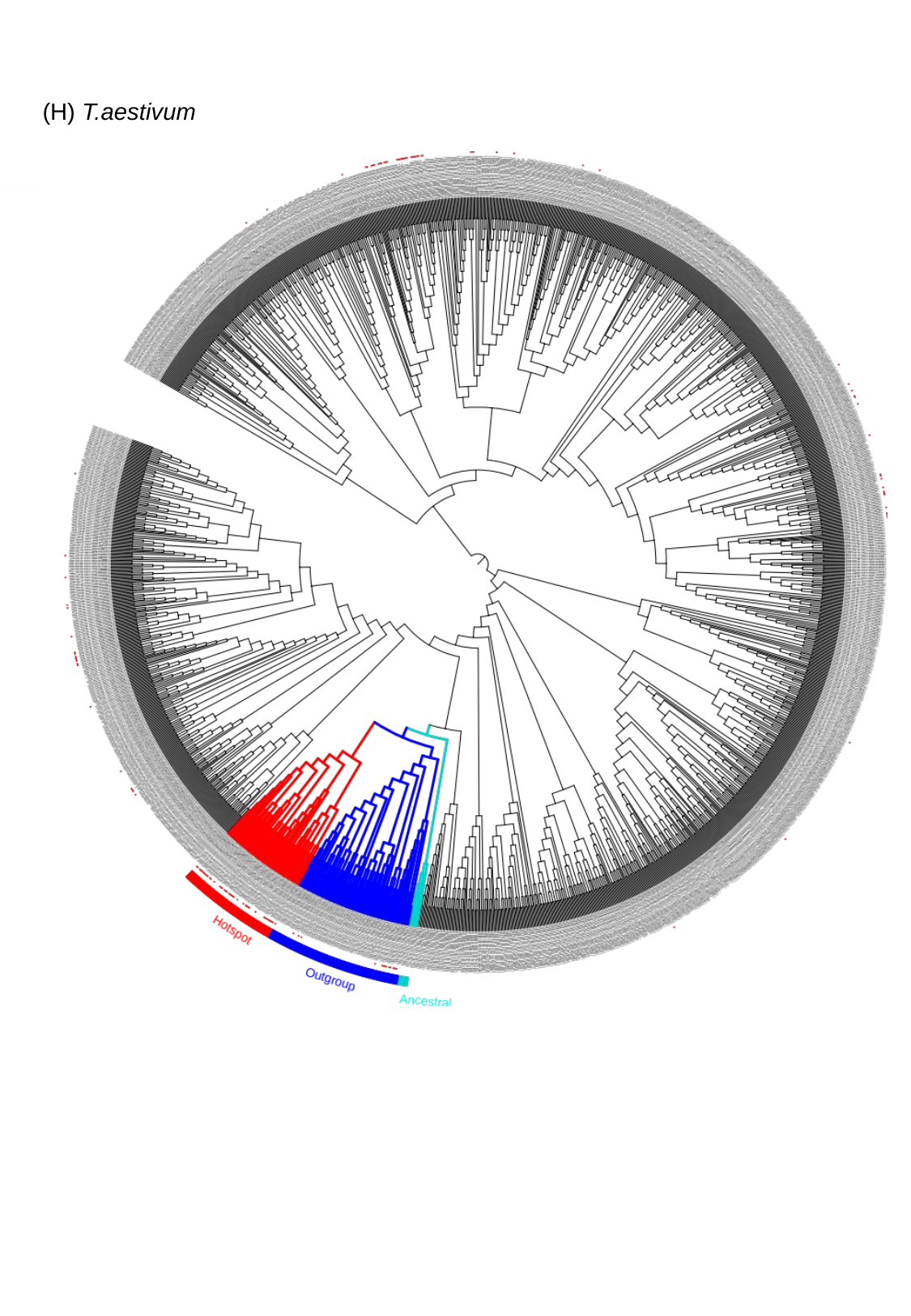

(H) T.aestivum

## Slide 9
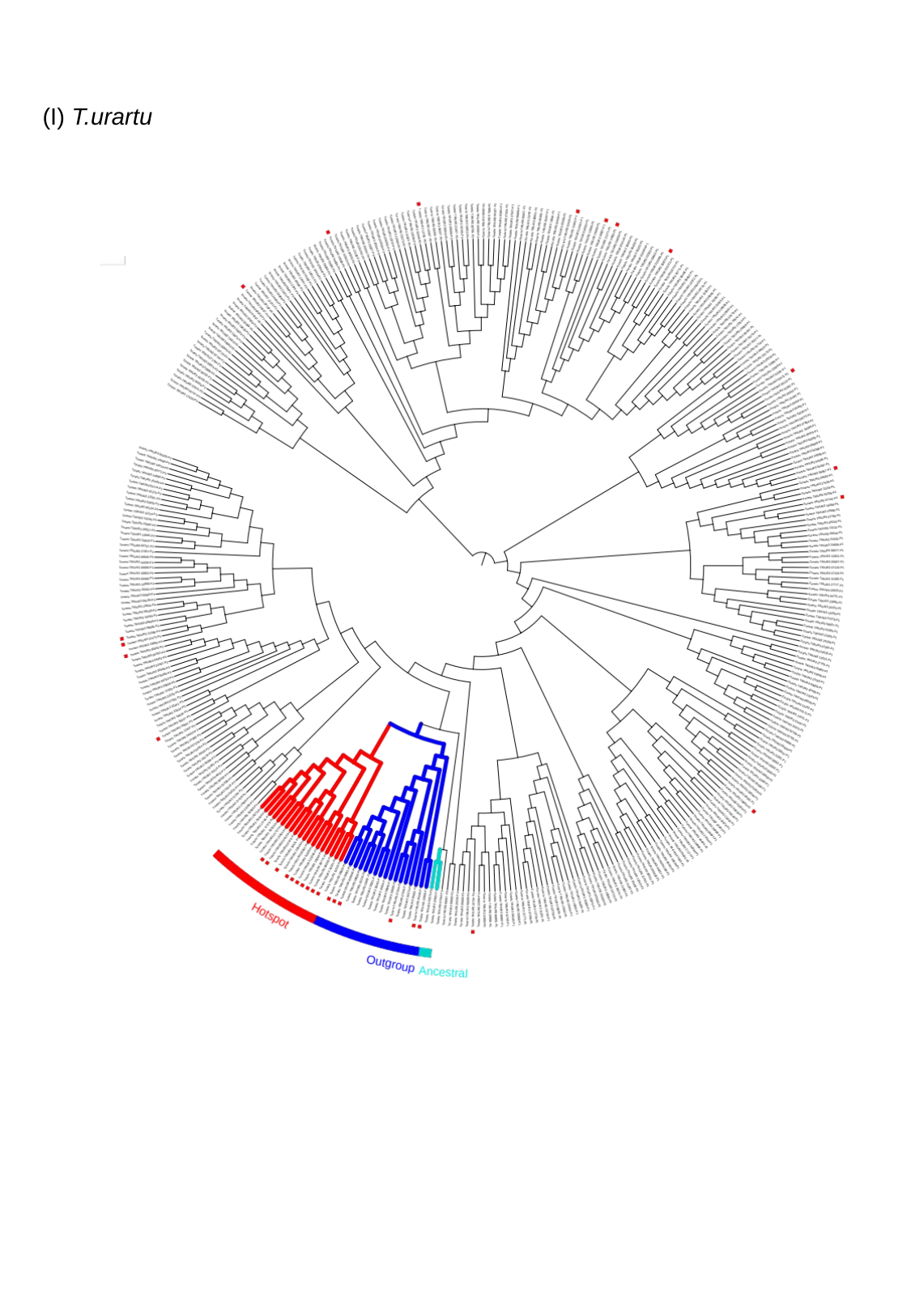

(I) T.urartu
