# Supplementary material for: Dominant integration locus drives continuous diversification of plant immune receptors with exogenous domain fusions
Source: Genome Biol. 2018 Feb 19;19:23. doi: 10.1186/s13059-018-1392-6 (PMC5819176; doi:10.1186/s13059-018-1392-6)
Supplement: Supplementary file 5 — Manual curation of the genomic regions surrounding MIC1 genes in Brachypodium and rice. (A) MIC1 NLR-IDs and surrounding genes. (B) Additional microsynteny analyses between rice and B. distachyon. (PPTX 150 kb) [file 13059_2018_1392_MOESM5_ESM.pptx]

## Slide 1
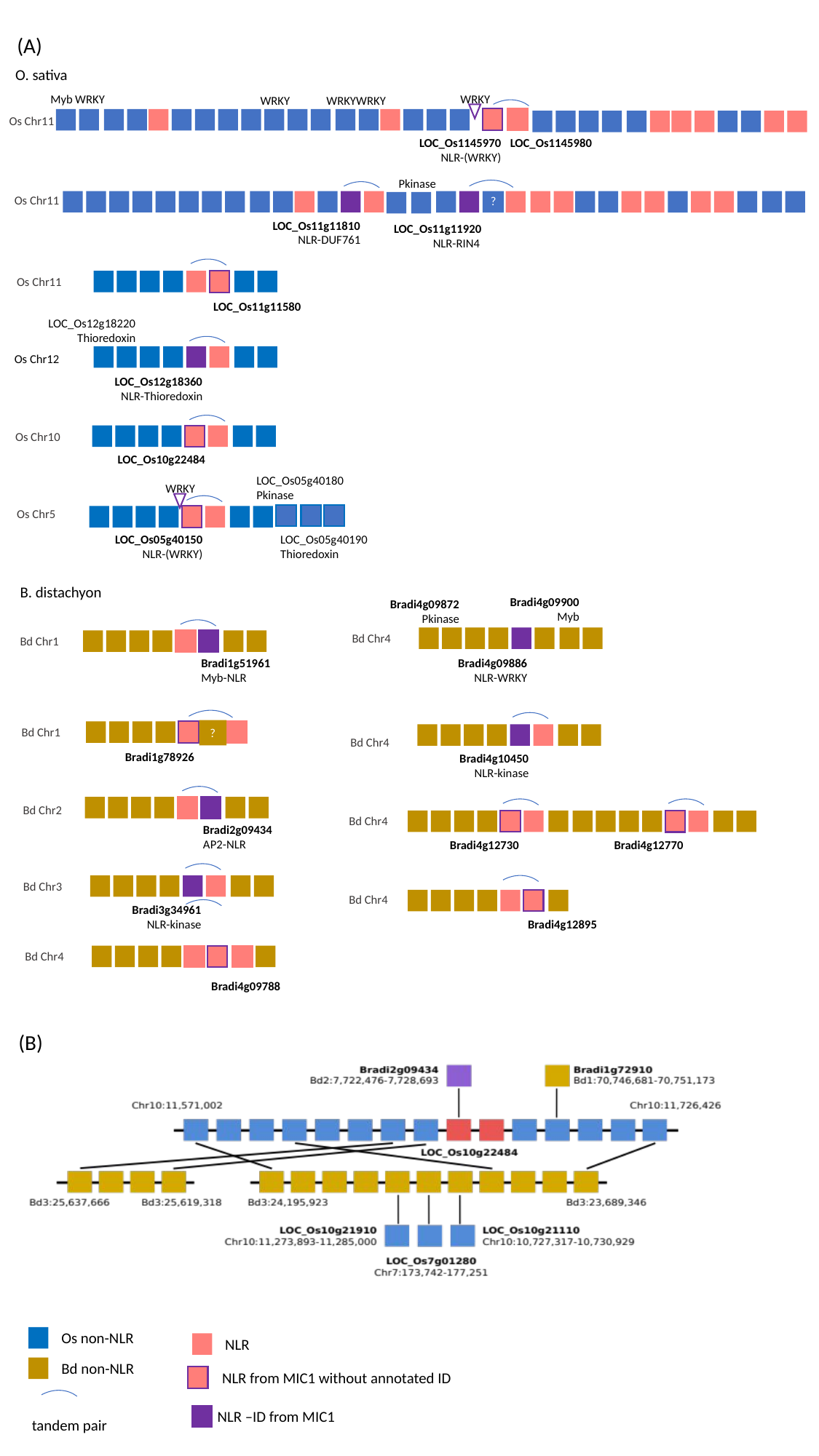

(A)
O. sativa
Myb
WRKY
WRKY
WRKY
WRKY
WRKY
Os Chr11
LOC_Os1145970
NLR-(WRKY)
LOC_Os1145980
Pkinase
Os Chr11
?
LOC_Os11g11810
LOC_Os11g11920
NLR-DUF761
NLR-RIN4
Os Chr11
LOC_Os11g11580
LOC_Os12g18220
Thioredoxin
Os Chr12
LOC_Os12g18360
NLR-Thioredoxin
Os Chr10
LOC_Os10g22484
LOC_Os05g40180
Pkinase
WRKY
Os Chr5
LOC_Os05g40190
Thioredoxin
LOC_Os05g40150
NLR-(WRKY)
B. distachyon
Bradi4g09900
Myb
Bradi4g09872
Pkinase
Bd Chr4
Bd Chr1
Bradi1g51961
Myb-NLR
Bradi4g09886
NLR-WRKY
Bd Chr1
?
Bd Chr4
Bradi1g78926
Bradi4g10450
NLR-kinase
Bd Chr2
Bd Chr4
Bradi2g09434
AP2-NLR
Bradi4g12730
Bradi4g12770
Bd Chr3
Bd Chr4
Bradi3g34961
NLR-kinase
Bradi4g12895
Bd Chr4
Bradi4g09788
(B)
Os non-NLR
NLR
Bd non-NLR
NLR from MIC1 without annotated ID
NLR –ID from MIC1
tandem pair
