# Supplementary figures and images for: Dominant integration locus drives continuous diversification of plant immune receptors with exogenous domain fusions
Source: Genome Biol. 2018 Feb 19;19:23. doi: 10.1186/s13059-018-1392-6 (PMC5819176; doi:10.1186/s13059-018-1392-6)

Motif I06

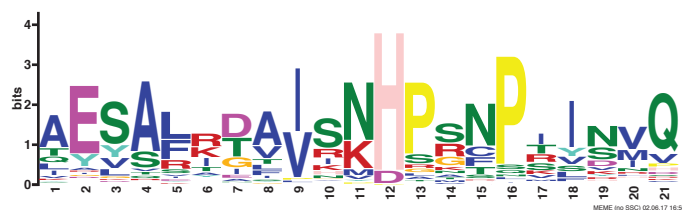

Motif I07

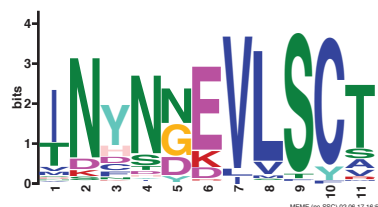

Motif I08

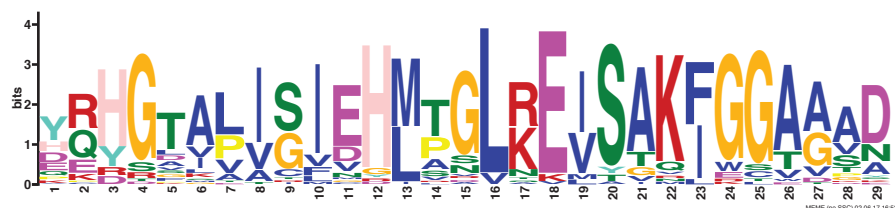

Motif I09

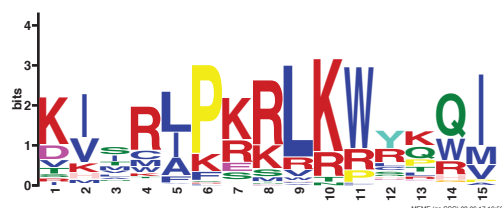

Motif I11

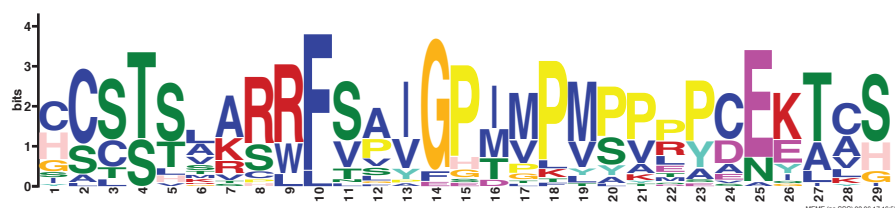

Motif I17

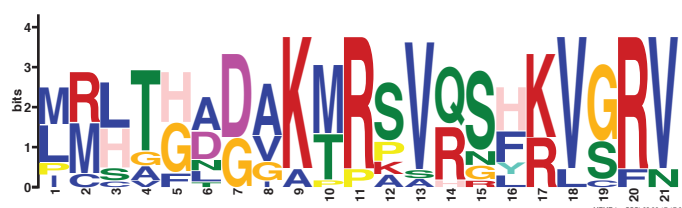

Motif I40

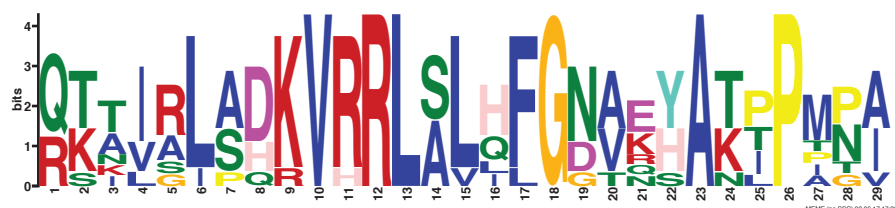

Supplement: Supplementary file 6 — Motifs identified using MEME that are associated with MIC1 clade. (PDF 866 kb) [file 13059_2018_1392_MOESM6_ESM.pdf]

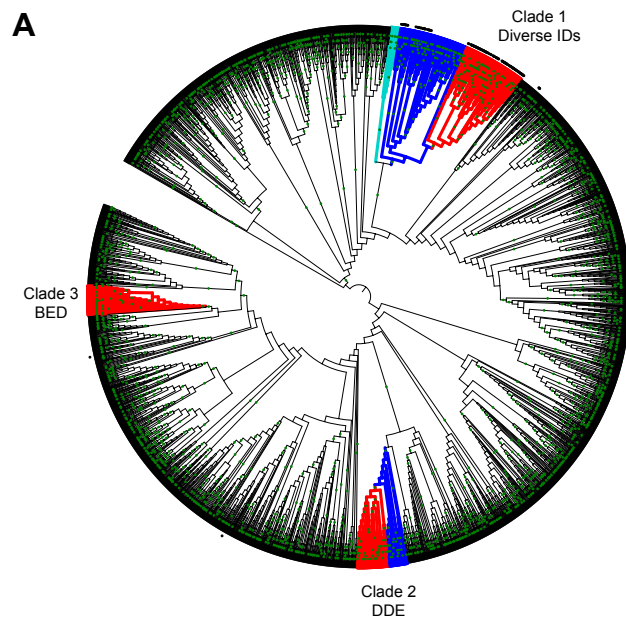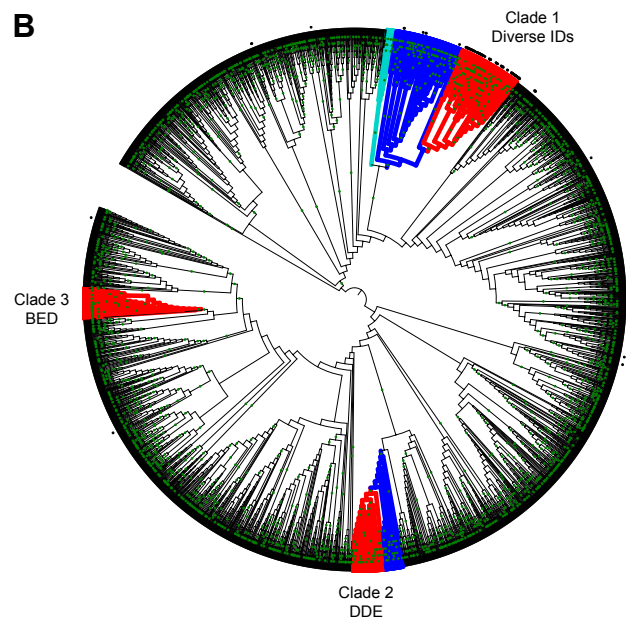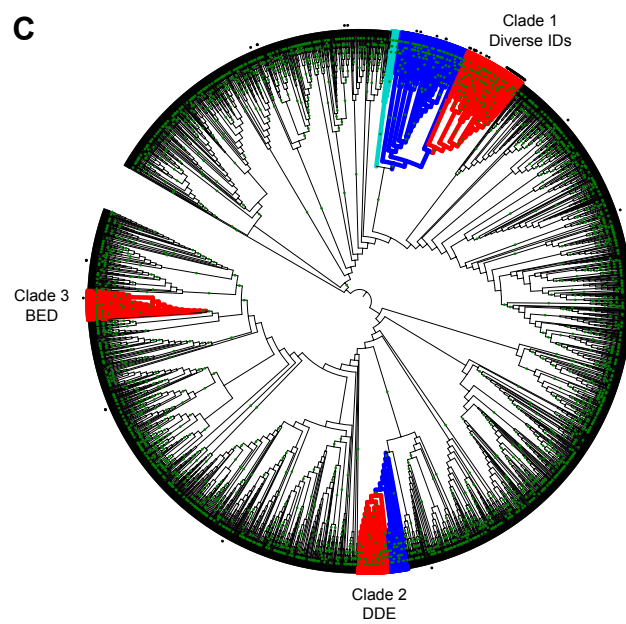

Supplement: Supplementary file 9 — Presence/absence of motifs (marked as black dots) relative to the NB phylogenetic tree. (A) I06, (B) I09, and (C) I11. (PDF 2852 kb) [file 13059_2018_1392_MOESM9_ESM.pdf]

A NB-ARC tree - MYB-AP2 domain swap

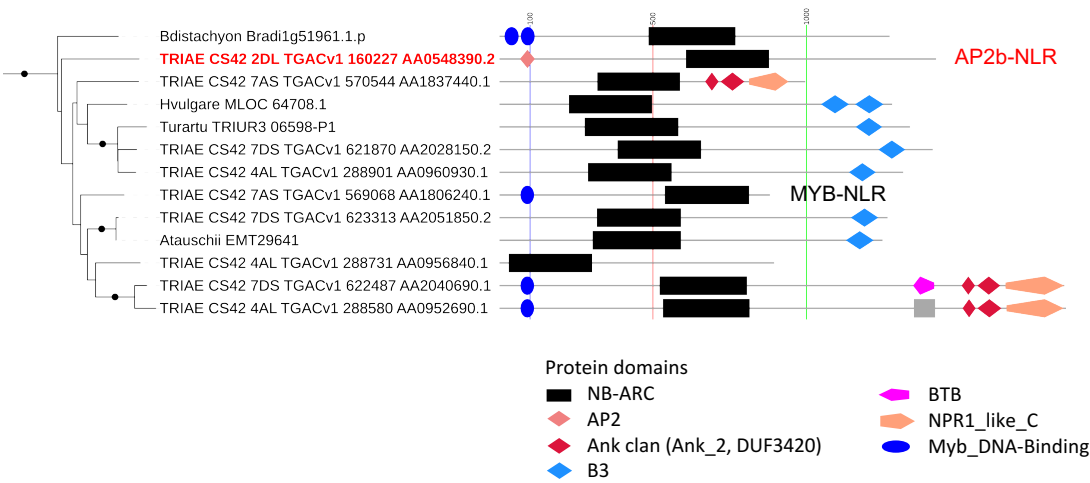

B AP2/ERF tree

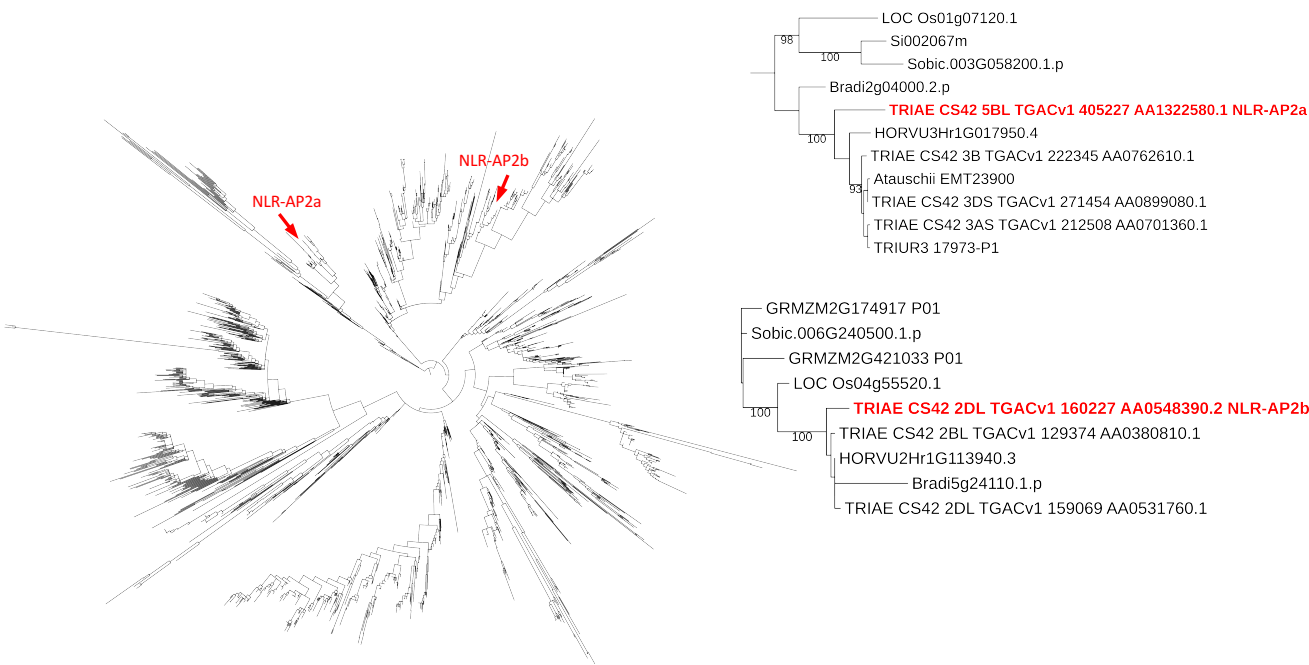

C

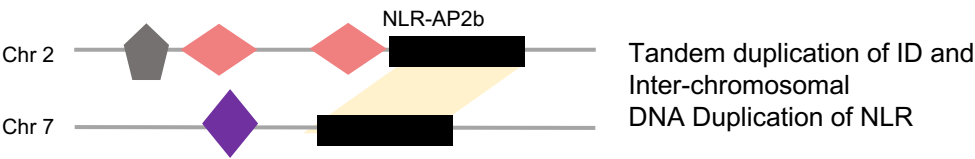

Supplement: Supplementary file 12 — The AP2b/MYB-NLR domain shuffling includes duplication of AP2 gene and inter-chromosomal transfer of NLR. (PDF 2232 kb) [file 13059_2018_1392_MOESM12_ESM.pdf]
