# Supplementary material for: Dominant integration locus drives continuous diversification of plant immune receptors with exogenous domain fusions
Source: Genome Biol. 2018 Feb 19;19:23. doi: 10.1186/s13059-018-1392-6 (PMC5819176; doi:10.1186/s13059-018-1392-6)
Supplement: Supplementary file 10 — Maximum likelihood phylogeny for eight gene families containing proteins with ID domains that were used to identify potential donor genes within each family for T. aestivum NLR-ID genes from the MIC1 clade. The gene identifiers highlighted in red are the acceptor genes containing an NB-ARC domain. (A) AP2/ERF family, (B) Exo70 family, (C) GRAS family, (D) Kelch_1 family, (E) NPR1_like_C, (F) Pkinase, (G) Pkinase_Tyr, and (H) WRKY. (PPTX 9868 kb) [file 13059_2018_1392_MOESM10_ESM.pptx]

## Slide 1
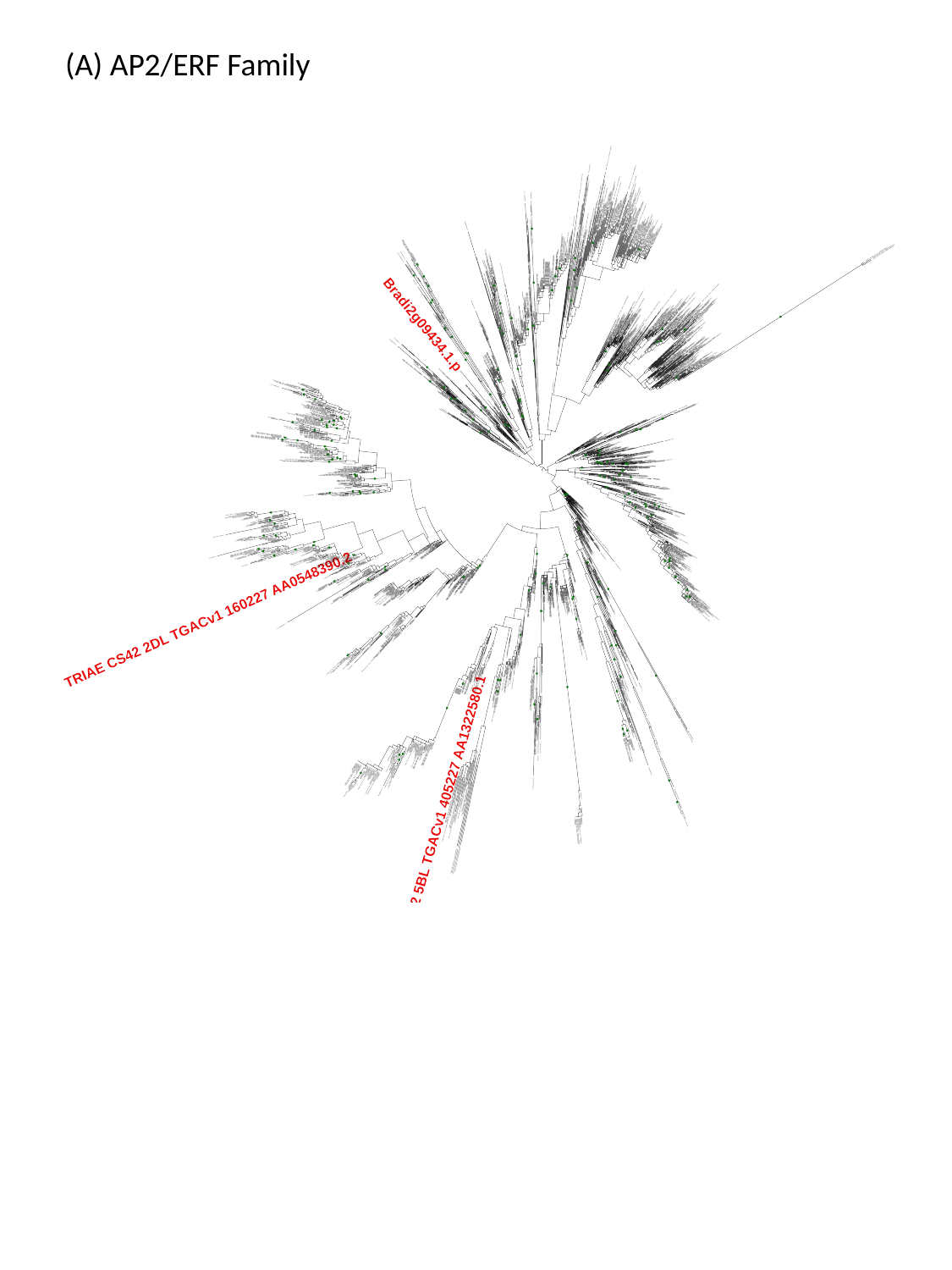

(A) AP2/ERF Family

## Slide 2
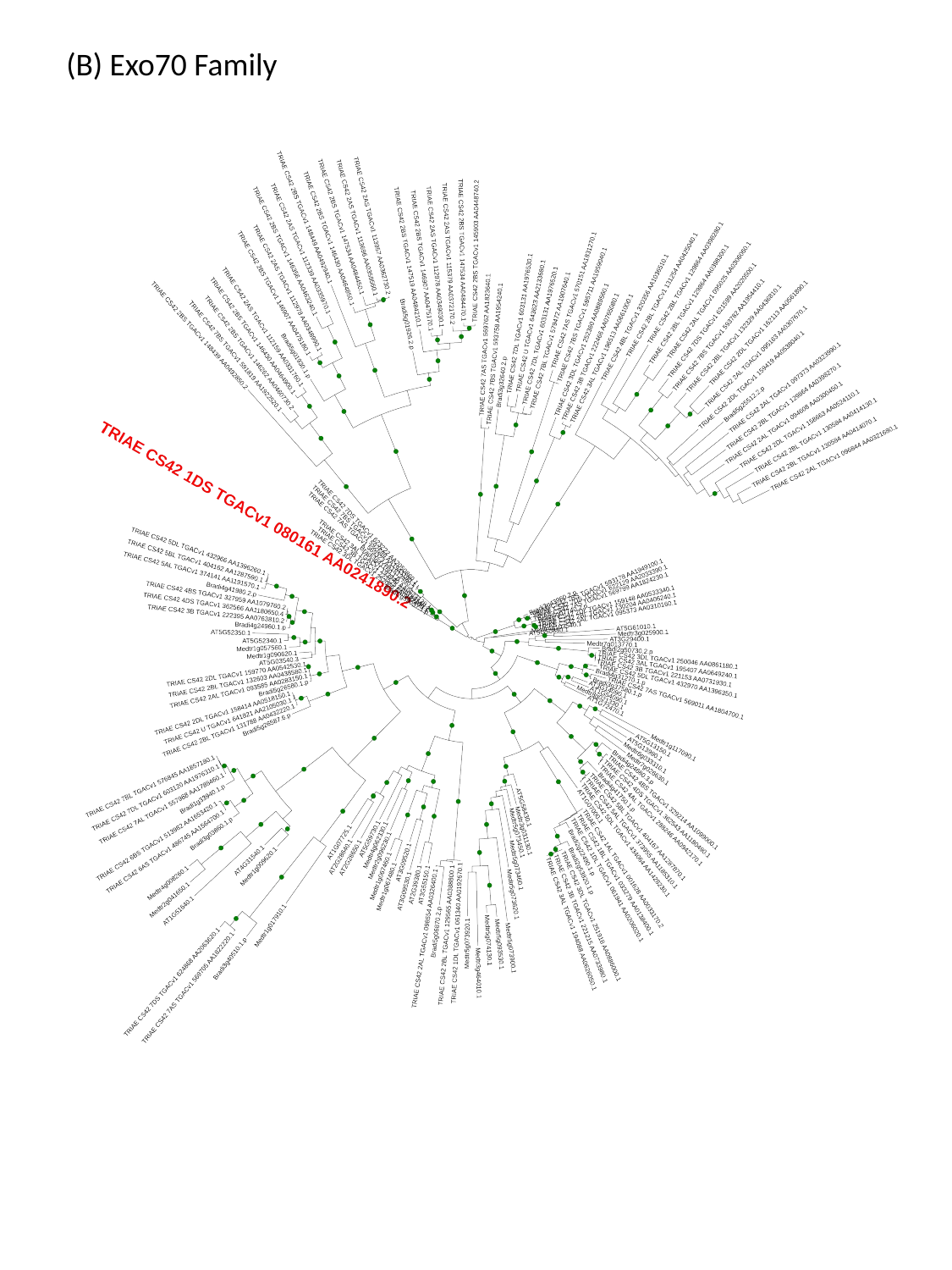

(B) Exo70 Family

## Slide 3
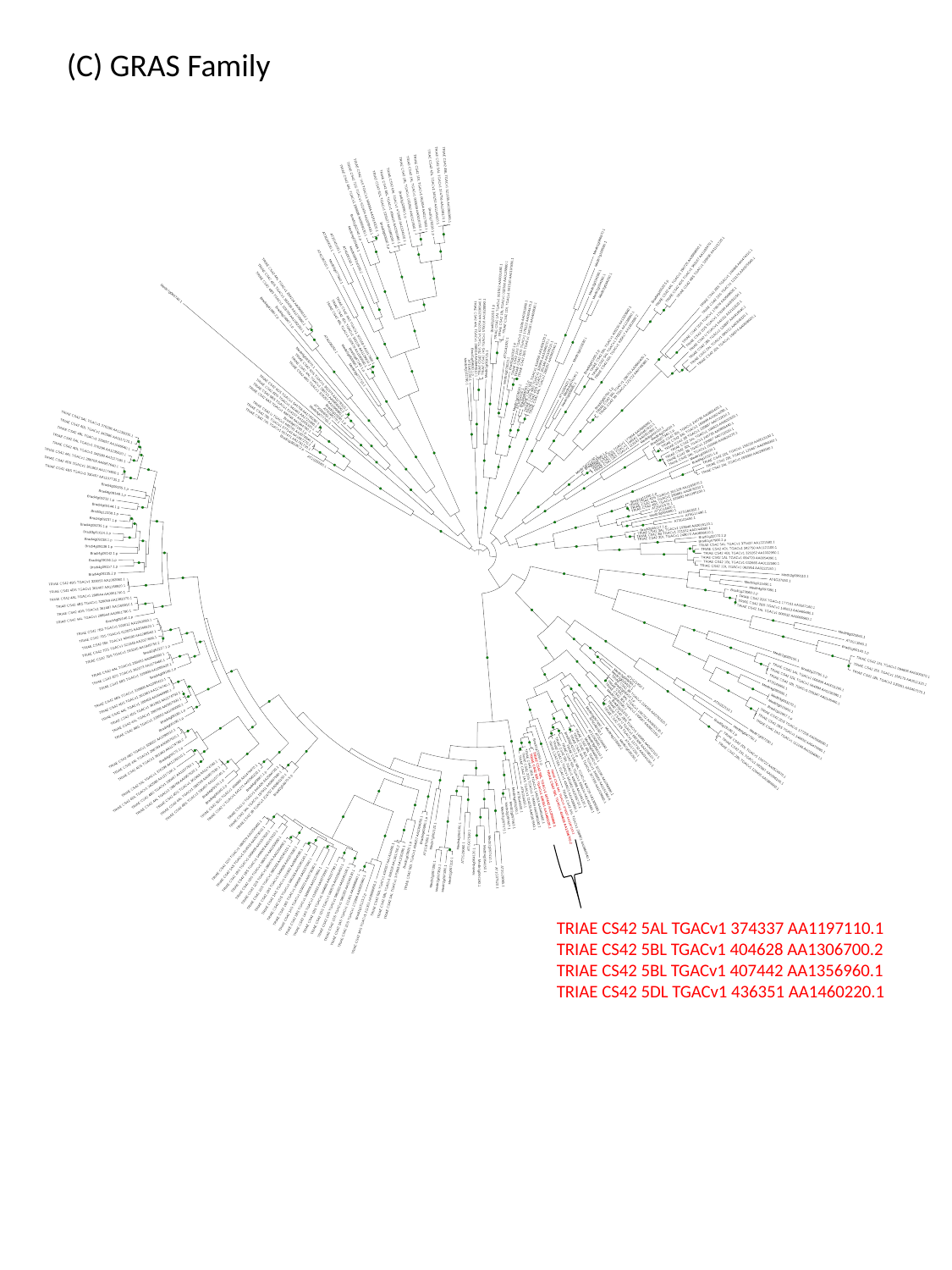

(C) GRAS Family
TRIAE CS42 5AL TGACv1 374337 AA1197110.1
TRIAE CS42 5BL TGACv1 404628 AA1306700.2
TRIAE CS42 5BL TGACv1 407442 AA1356960.1
TRIAE CS42 5DL TGACv1 436351 AA1460220.1

## Slide 4
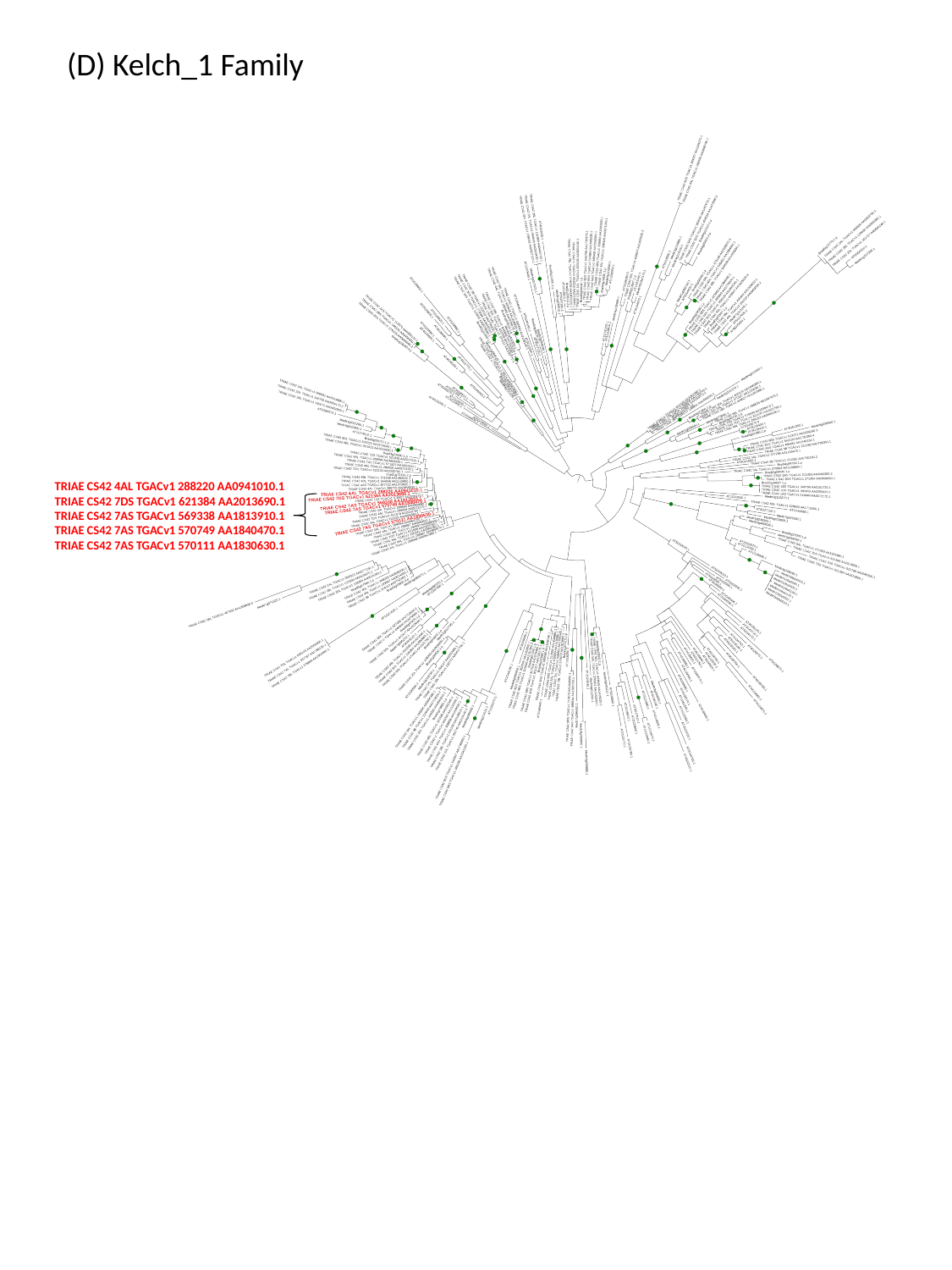

(D) Kelch_1 Family
TRIAE CS42 4AL TGACv1 288220 AA0941010.1
TRIAE CS42 7DS TGACv1 621384 AA2013690.1
TRIAE CS42 7AS TGACv1 569338 AA1813910.1
TRIAE CS42 7AS TGACv1 570749 AA1840470.1
TRIAE CS42 7AS TGACv1 570111 AA1830630.1

## Slide 5
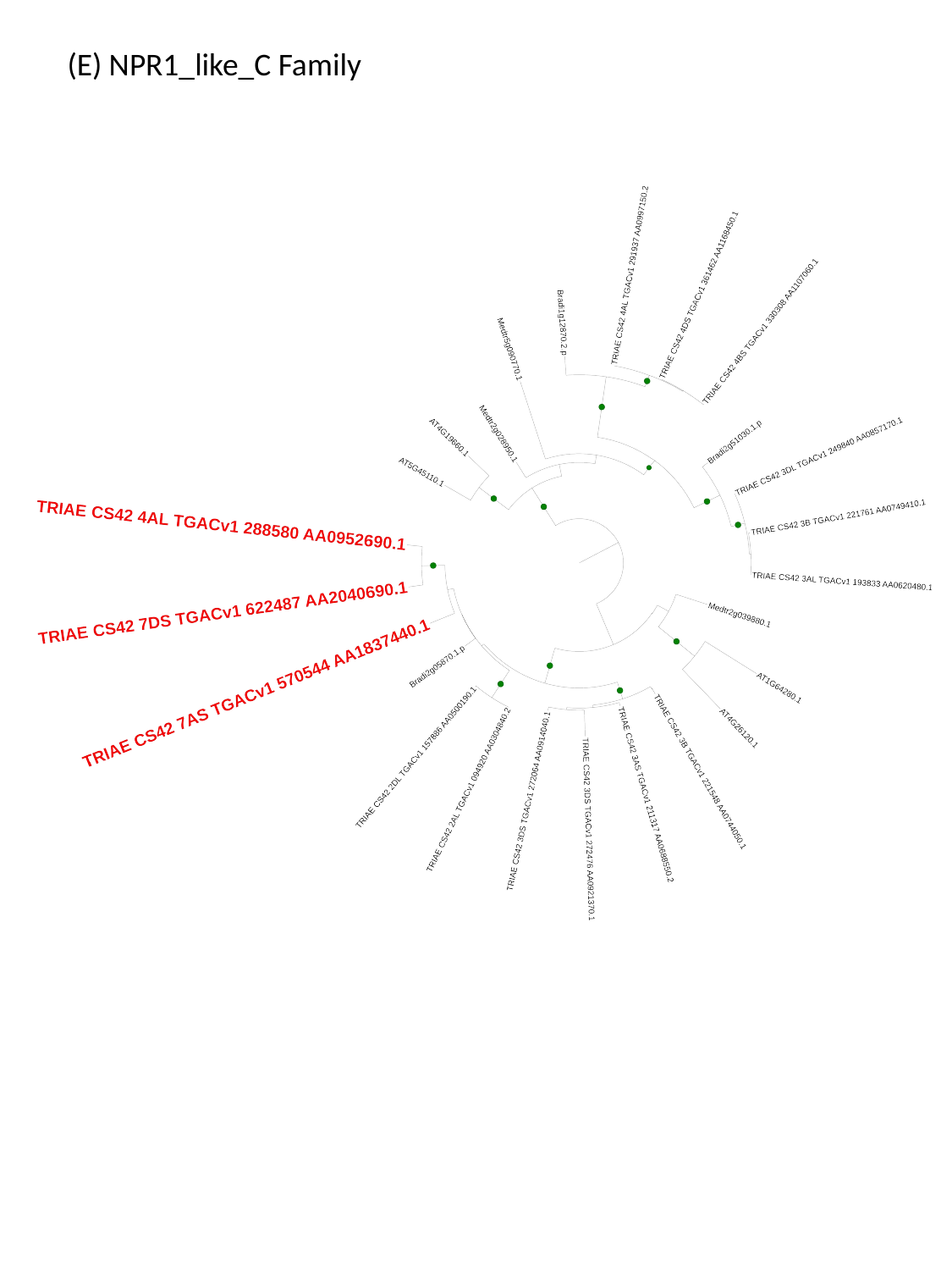

(E) NPR1_like_C Family

## Slide 6
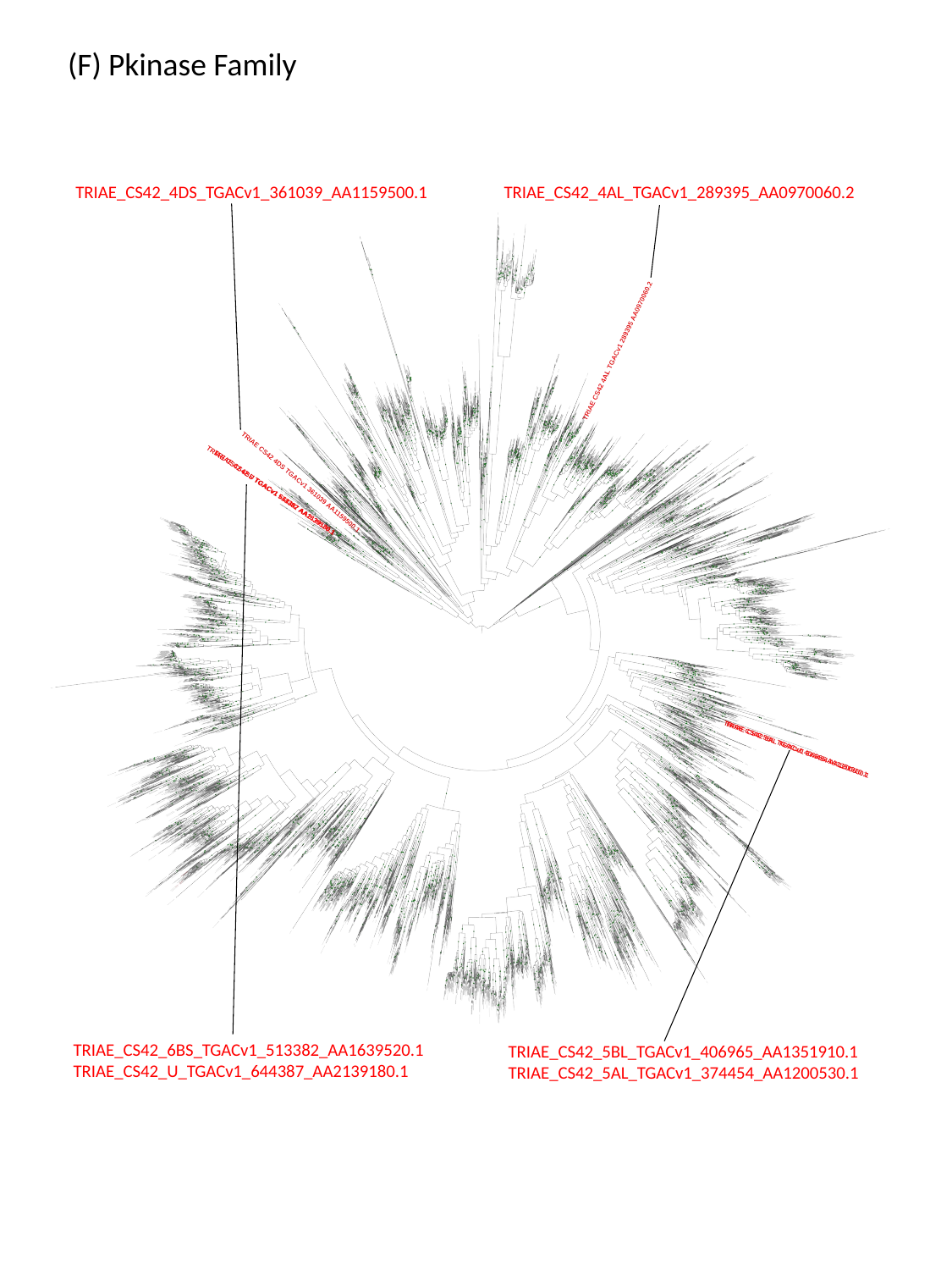

(F) Pkinase Family
TRIAE_CS42_4AL_TGACv1_289395_AA0970060.2
TRIAE_CS42_4DS_TGACv1_361039_AA1159500.1
TRIAE_CS42_6BS_TGACv1_513382_AA1639520.1
TRIAE_CS42_U_TGACv1_644387_AA2139180.1
TRIAE_CS42_5BL_TGACv1_406965_AA1351910.1
TRIAE_CS42_5AL_TGACv1_374454_AA1200530.1

## Slide 7
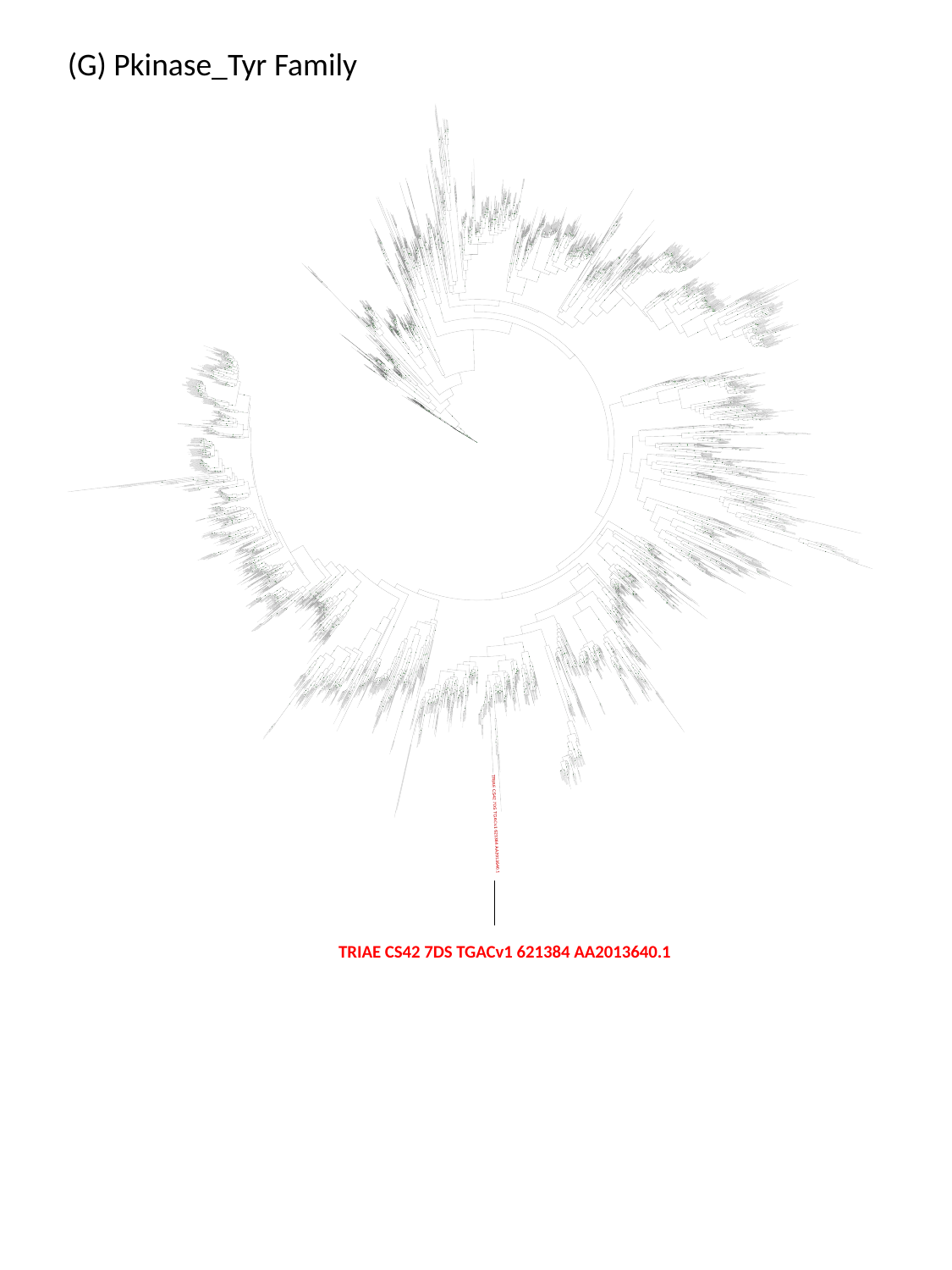

(G) Pkinase_Tyr Family
TRIAE CS42 7DS TGACv1 621384 AA2013640.1

## Slide 8
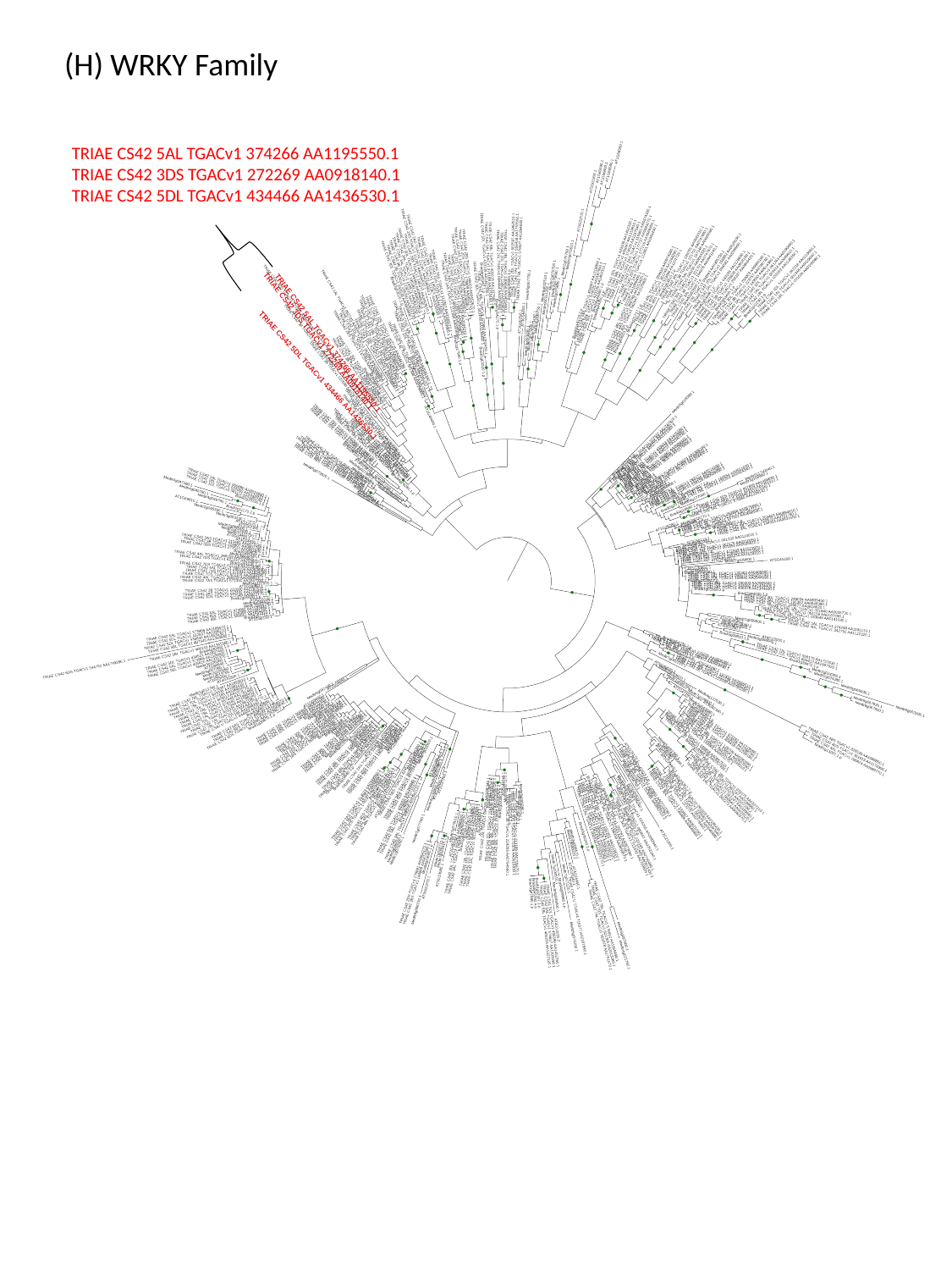

(H) WRKY Family
TRIAE CS42 5AL TGACv1 374266 AA1195550.1
TRIAE CS42 3DS TGACv1 272269 AA0918140.1
TRIAE CS42 5DL TGACv1 434466 AA1436530.1
