# Supplementary material for: Dominant integration locus drives continuous diversification of plant immune receptors with exogenous domain fusions
Source: Genome Biol. 2018 Feb 19;19:23. doi: 10.1186/s13059-018-1392-6 (PMC5819176; doi:10.1186/s13059-018-1392-6)
Supplement: Supplementary file 13 — Bar plot of number of NLRs in each clade and percent of NLRs from each clade that overlap with predicted Helitrons. (PDF 10 kb) [file 13059_2018_1392_MOESM13_ESM.pdf]

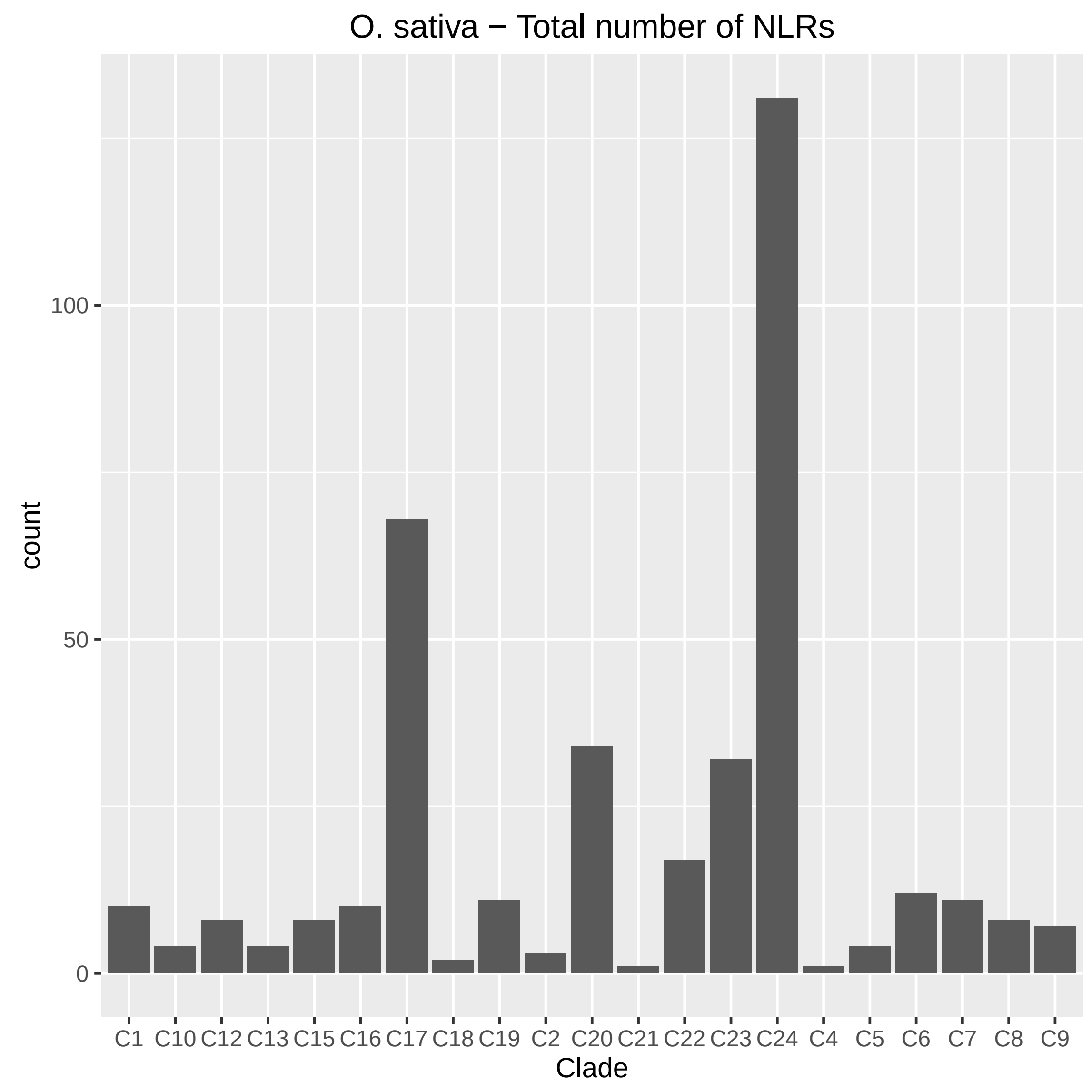
O. sativa − Total number of NLRs
count
0
50
100
C1 C10 C12 C13 C15 C16 C17 C18 C19 C2 C20 C21 C22 C23 C24 C4 C5 C6 C7 C8 C9
Clade

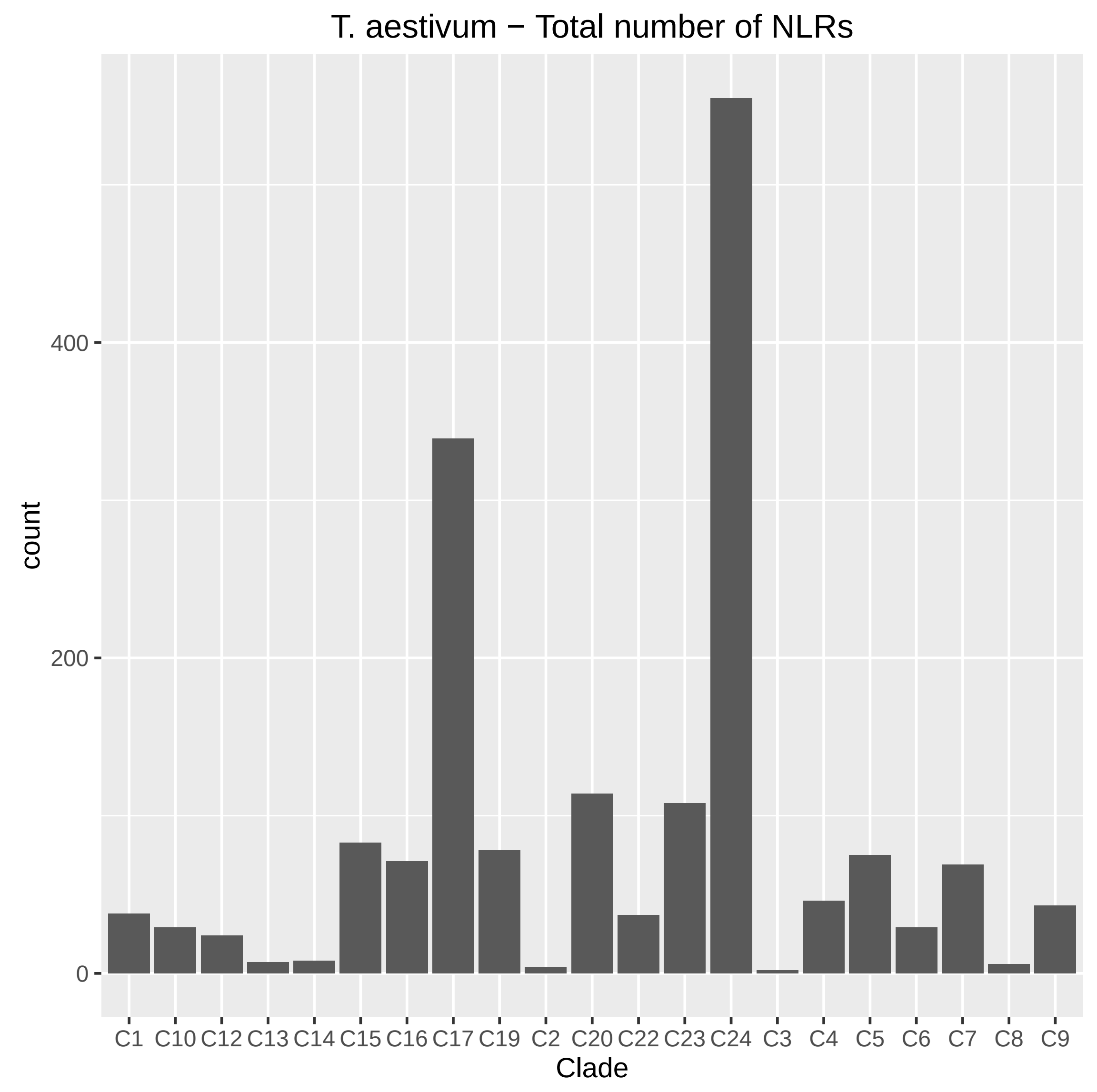
T. aestivum − Total number of NLRs
count
0
200
400
C1 C10 C12 C13 C14 C15 C16 C17 C19 C2 C20 C22 C23 C24 C3 C4 C5 C6 C7 C8 C9
Clade

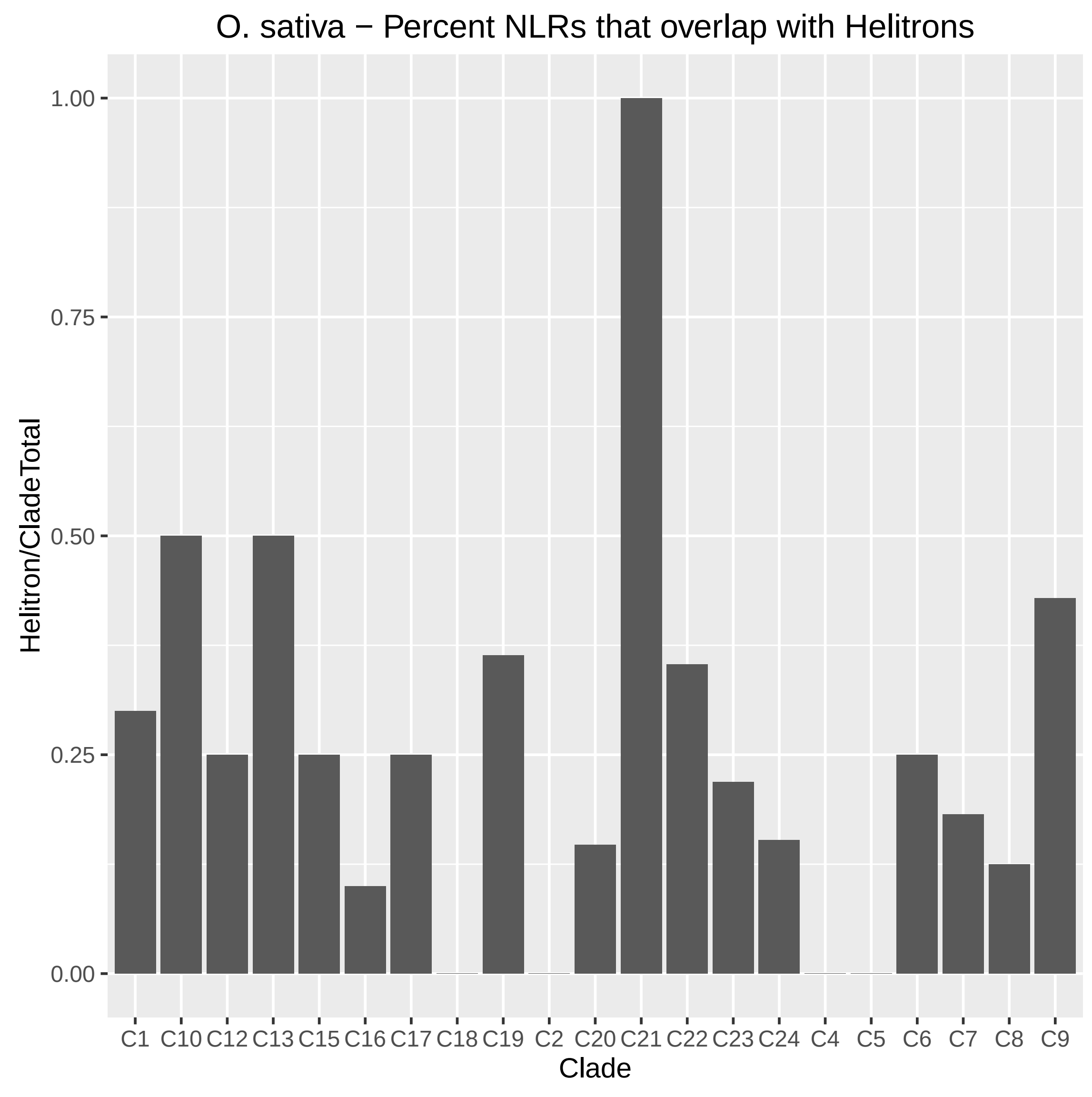
O. sativa − Percent NLRs that overlap with Helitrons
Helitron/CladeTotal
0.00
0.25
0.50
0.75
1.00
C1 C10 C12 C13 C15 C16 C17 C18 C19 C2 C20 C21 C22 C23 C24 C4 C5 C6 C7 C8 C9
Clade

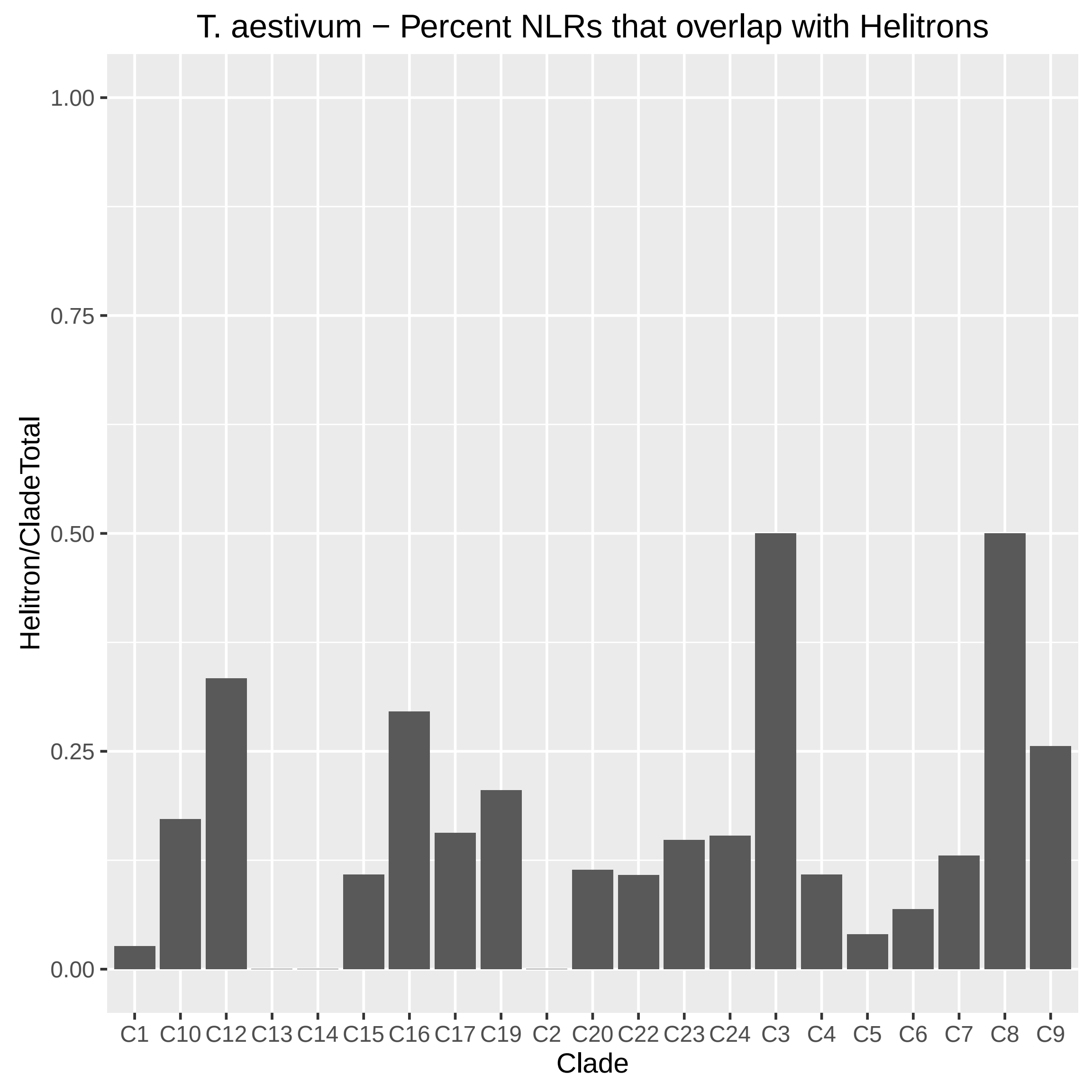
T. aestivum − Percent NLRs that overlap with Helitrons
Helitron/CladeTotal
0.00
0.25
0.50
0.75
1.00
C1 C10 C12 C13 C14 C15 C16 C17 C19 C2 C20 C22 C23 C24 C3 C4 C5 C6 C7 C8 C9
Clade
